# DeepExoMir: A Reproducible RNA Language Model Framework for CLIP-Seq-Supported MicroRNA Target-Site Prioritization

**DOI:** 10.3390/ijms27146184

**Published:** 2026-07-10

**Authors:** Wen-Hsien Lin, Chia-Ni Hsiung, Wen-Yu Lien, Martin Sieber

**Affiliations:** 1 AI and Data Applications Division, GGA Corp., Taipei 114065, Taiwan; 2 BIONET Therapeutics Corp., Taipei 114065, Taiwan; 3 BIONET Corp., Taipei 114065, Taiwan

**Keywords:** microRNA target prediction, deep learning, RNA language models, CLIP-seq, benchmark, ablation study, melanogenesis, exosomal microRNA, molecular informatics, reproducibility

## Abstract

MicroRNAs regulate gene expression post-transcriptionally, yet target prediction faces a credibility gap: published methods drop sharply against CLIP-seq-validated negatives. We present DeepExoMir, a deep learning framework integrating frozen RiNALMo RNA language model embeddings with biologically informed features. Under a dual-probe ablation protocol on three miRBench test sets, DeepExoMir reaches mean AU-PRC 0.855, surpassing eight retrained baselines (paired bootstrap p<0.001). Evolutionary conservation and duplex structure prove largely redundant with language-model priors, motivating a structure-free Lite variant (0.863). On nine exosomal miRNAs from a companion melanogenesis study, DeepExoMir recovers literature-validated targets and ranks canonical pigmentation regulators (KITLG, MITF, TYRP1) in the top 5%.

## 1. Introduction

### 1.1. The Rigorous Evaluation Crisis in miRNA Target Prediction

**Figure 1:**
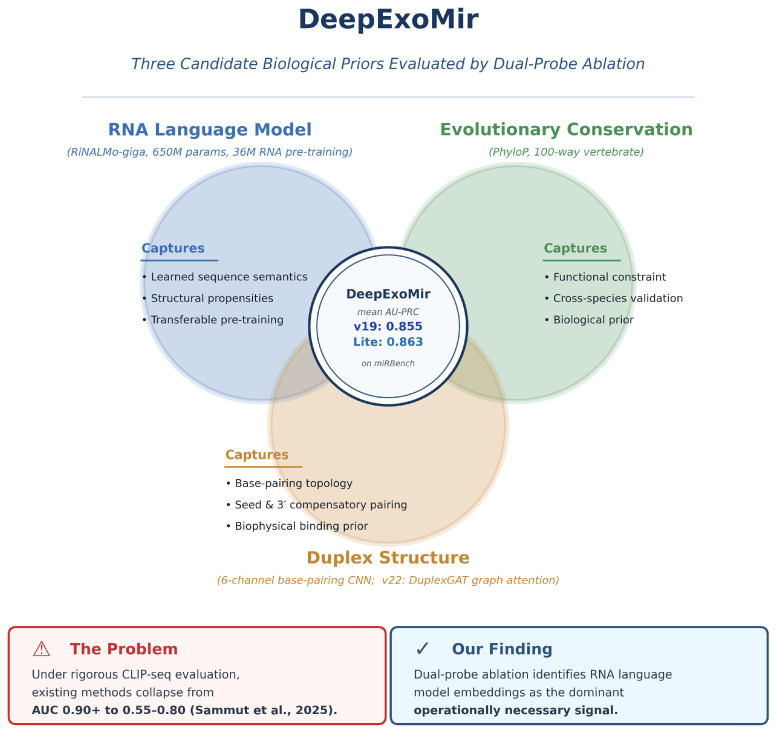
Three candidate biological priors evaluated under the dual-probe ablation protocol. DeepExoMir v19 combines three input streams—RNA language model embeddings (RiNALMo-giga), evolutionary conservation (PhyloP), and explicit base-pairing geometry (six-channel BP CNN; an exploratory v22 variant adds DuplexGAT)—and is evaluated on three miRBench CLIP-seq test sets where v19 reaches mean AU-PRC 0.855 and outperforms all eight retrained baselines (paired bootstrap p<0.001). **Dual-probe ablation identifies RiNALMo as the dominant operationally necessary signal**; inference-time feature masking (Figure 3A) and retrain-from-scratch ablations (Section 2.3) agree that PhyloP conservation and explicit structural features are largely redundant with RiNALMo’s implicit priors, motivating the simpler DeepExoMir-Lite variant (mean AU-PRC 0.863; Table 1). The graphic depicts the candidate-prior architecture and summarizes both the pre-specified v19 reference model (mean test AU-PRC 0.855) and the ablation-derived DeepExoMir-Lite variant (0.863) [4].

**Figure 2:**
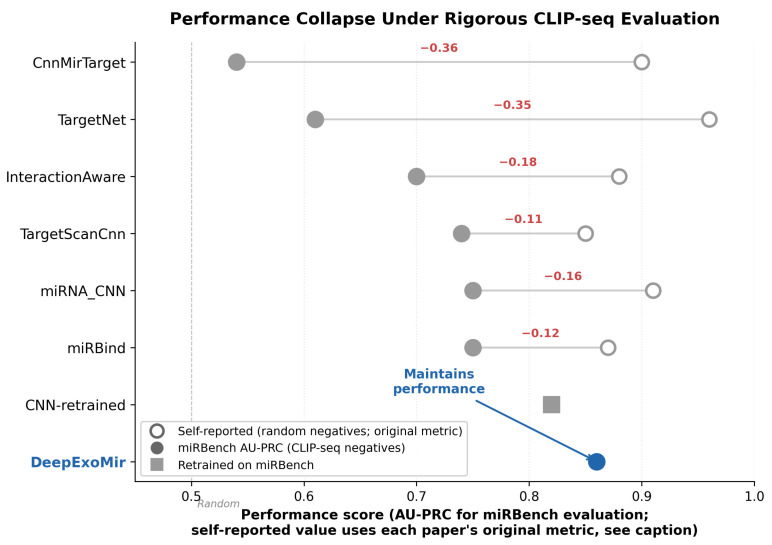
DeepExoMir resists the performance collapse that affects existing methods. Dumbbell plot comparing each method’s self-reported performance under random-negative evaluation (open circles; the original studies used various metrics including ROC-AUC, accuracy, or average precision) against the same methods re-evaluated on miRBench CLIP-seq-validated negatives in AU-PRC (filled circles, as reported by Sammut et al. [4]). Under rigorous evaluation, existing methods show substantially lower AU-PRC (drops shown in red), whereas DeepExoMir (blue) maintains consistent performance across the three miRBench test datasets at AU-PRC 0.86. The square marker for “CNN-retrained” represents the best CNN baseline retrained on the miRBench training data [4]; this baseline is the most stringent comparison reference (AU-PRC 0.82). The development-time progression of our model versions (cumulative addition of signal sources, ROC-AUC) is reported separately in Figure 3B and Table 2 to keep the cross-method benchmark message in this figure focused.

MicroRNA (miRNA) target prediction has long been considered a solved problem. We revisit that assumption with DeepExoMir, the framework overviewed in Figure 1. Deep learning methods routinely report performance values above 0.90: DeepMirTar [1] claimed 93.5% accuracy, TargetNet [2] reported ROC-AUC near 0.97, and miRBind [3] reported average precision (AU-PRC) above 0.87. Yet rigorous benchmarking by Sammut et al. [4] recently revealed that these published methods perform dramatically worse under evaluation conditions that better reflect biological reality. When tested against experimentally validated CLIP-seq negatives with bias-corrected miRNA frequency distributions, the same methods collapse to AU-PRC values of 0.55–0.80 (Figure 2). This substantial gap between each method’s self-reported performance and its miRBench AU-PRC under rigorous evaluation suggests that many existing architectures do not learn generalizable targeting principles but instead exploit artifacts of random negative sampling. Understanding which architectural principles survive rigorous evaluation is therefore a critical methodological question for the field.

### 1.2. Three Biological Signals Relevant to miRNA Targeting

miRNAs are short non-coding RNAs of approximately 22 nucleotides that regulate gene expression post-transcriptionally by guiding the RNA-induced silencing complex (RISC) to complementary sequences in target mRNAs [5]. The canonical targeting mechanism centers on the seed region (positions 2–8 from the 5’ end) forming Watson–Crick base pairs with complementary sites in the target 3’UTR [6], but functional targeting also involves 3’ compensatory pairing, centered sites, and non-canonical interactions [7,8,9]. Over 2600 mature human miRNAs [10] collectively regulate more than 60% of protein-coding genes [11], with dysregulation implicated in cancer, cardiovascular disease, and neurodegeneration [12].

Computational prediction exploits three broadly distinct signal sources. First, *sequence-level patterns* encode targeting rules learnable directly from data [13,14]—the basis for classical tools such as TargetScan [15] and miRDB [16] and for more recent foundation models such as RiNALMo [17], which was pre-trained on 36 million non-coding RNA sequences. Second, *evolutionary conservation* marks functionally important target sites through cross-species selection pressure, quantified by measures such as PhyloP [18]. Third, *duplex structure* determines biophysical binding feasibility through base-pairing topology, stacking interactions, and secondary structure accessibility [19]. Prior methods typically emphasize one or two of these signal sources but rarely integrate all three in a principled way.

### 1.3. This Work: Testing Whether Biological Priors Improve Robustness

Here we present DeepExoMir, a hybrid framework centered on pre-trained RNA language model embeddings and augmented with evolutionary-conservation and base-pairing-geometry features (Figure 1). Throughout, “miRNA target prediction” refers to the miRBench operational task [4]: discriminating CLIP-seq-supported binding sites from bias-corrected CLIP-seq negatives. Direct post-transcriptional repression is a distinct downstream question not addressed by this benchmark. We do not claim a priori that all three signal sources are independently necessary; rather, we *test whether combining complementary biological priors improves robustness under rigorous CLIP-seq evaluation*, and we report which priors matter at which stage of the training/inference cycle. DeepExoMir integrates (i) frozen RiNALMo embeddings, enabling one-time precomputation without task-specific fine-tuning; (ii) PhyloP evolutionary conservation features; and (iii) explicit base-pairing geometry encoded via a six-channel base-pairing CNN (main model v19). An extended exploratory variant (v22) additionally incorporates a duplex graph attention network (DuplexGAT) as a proof-of-concept module for graph-based duplex modeling; v22 is *not* the primary contribution of this work and is reported alongside, not ahead of, v19. Headline benchmarks and retrospective validation below use the main v19 model.

**Figure 3:**
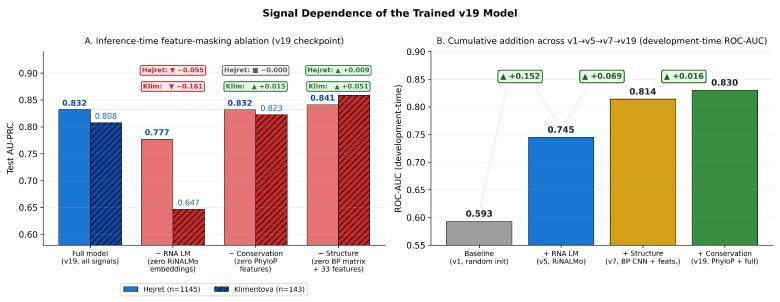
Signal dependence: complementary during development, RiNALMo-dominant at inference. (**A**) *Inference-time feature-masking* ablation on the Hejret CLIP-seq test set (n=965). Zeroing the RiNALMo per-token embeddings reduces AU-PRC by 0.055 (from 0.832 to 0.777); zeroing PhyloP conservation features has negligible effect (−0.0002); zeroing the entire structural-feature vector and base-pairing matrix does not reduce performance (+0.009 AU-PRC, within noise). Similar patterns hold on Klimentová (RNA LM −0.161; PhyloP +0.015; structure +0.051). In the Δ-vs-FULL chips, ▴, ▾, and ▪ denote an increase, a decrease, and a negligible change (|Δ|≤0.005) in AU-PRC, respectively. The large RNA-LM effect and near-zero effects for the other two signals describe the trained model’s dependency at its final operating point—they do not establish that conservation or structure are uninformative in principle. The retrain-from-scratch ablations reported in Section 2.3 (Table 3) extend this analysis by quantifying what the model could have learned in the absence of each signal. (**B**) *Conceptual cumulative-addition view* of the three signal sources, shown in the order they were incorporated during model development: baseline encoder, + RNA language model embeddings, + explicit structural features, and + evolutionary conservation (metric: development-time ROC-AUC). The plotted values summarize representative development-time checkpoints associated with these successive signal additions. The largest single jump (Δ≈+0.15 ROC-AUC) coincides with adding the pre-trained RNA language model; subsequent additions of structural and conservation features contributed smaller, consistent incremental gains. Complete per-version milestone values (v1, v8, v10+, v14-2L, v19, etc.) are reported separately in Table 2. Together panels (**A**,**B**) support the interpretation that RiNALMo pre-trained embeddings are the principal signal of this model; conservation and structural features helped during training but provide largely overlapping information at inference. This divergence between development-time and inference-time attribution is characteristic of highly over-parameterized encoders.

On three independent miRBench test datasets with CLIP-seq-validated negatives, DeepExoMir reaches mean AU-PRC 0.855 and outperforms all eight retrained miRBench baselines at paired sample-level bootstrap p<0.001 (Table 1). To assess which of the three candidate signals is actually needed, we apply a *dual-probe ablation protocol*: (i) inference-time feature masking on the v19 checkpoint, and (ii) retrain-from-scratch ablations in which each signal is zeroed at every sample during both training and evaluation. The two probes return the same ranking. RiNALMo is the dominant and operationally necessary signal (inference-time AU-PRC drop 0.055–0.161; retrain-from-scratch mean drop 0.029). PhyloP conservation and explicit structural features are largely redundant with RiNALMo’s implicit priors (inference-time Δ within noise; retrain-from-scratch deltas −0.004 and +0.008). Attention analysis adds an interpretability signal consistent with canonical seed-region biology, not a mechanistic proof.

For practical use beyond benchmark datasets, we ran retrospective validation against nine experimentally characterized exosomal miRNAs from our companion study on melanogenesis regulation (Hsiung et al., 2026 [20]). Because exosomal miRNAs can be transferred between cells and regulate gene expression in recipient cells [21,22], target prediction for these miRNAs is biologically meaningful. We introduce an explicit four-tier training-data leakage disclosure: five of the nine miRNAs (all novel discoveries from the companion study) occur ≤6 times in our 1.9 M training pairs and are effectively unseen. On these novel miRNAs, TargetScan 8.0 provides zero predictions (a consequence of its database cutoff), whereas DeepExoMir generates thousands of ranked candidate predictions per miRNA and prioritizes canonical melanogenesis regulators at meaningful percentile ranks. All evidence presented in this retrospective validation is *indirect* (rank-based, literature overlap, pathway enrichment); direct target-level functional validation is deferred to future wet-lab work.

**Table 1:** Performance comparison on miRBench benchmark (AU-PRC). All eight paired-bootstrap baselines use the retrained/pre-supplied checkpoints from the miRBench Python package [4]; DeepExoMir v19 and all baselines were evaluated under identical negative-sampling conditions on the three held-out test datasets. Paired sample-level bootstrap (10,000 resamples for Hejret/Klimentová, 2000 for Manakov) computes the AU-PRC difference. **Δ vs. v19 is defined as (method mean AU-PRC) − (v19 mean AU-PRC)**; baselines therefore show negative values, and DeepExoMir-Lite a small positive value. Significance: *** p<0.001. Methods sorted by mean AU-PRC, descending. For reference only, Sammut et al. [4] additionally report a best-case retrained CNN baseline (not included in the miRBench package’s predictor API) achieving mean AU-PRC ∼0.82; DeepExoMir’s mean of 0.855 therefore represents a ∼0.04 AU-PRC gain over this literature-reported retrained CNN benchmark.

Method	Hejret	Klim.	Manakov	Mean	Δ vs. v19
**DeepExoMir v19** (pre-specified reference)	**0.848**	**0.868**	**0.848**	**0.855**	reference
**DeepExoMir-Lite** (ablation-derived)	0.866	0.871	0.853	0.863	+0.008 ^†^
TargetScanCnn [23]	0.711	0.745	0.774	0.743	−0.112 ***
miRNA_CNN (Hejret) [24]	0.773	0.739	0.711	0.741	−0.114 ***
InteractionAware [25]	0.741	0.664	0.692	0.699	−0.156 ***
Seed6mer	0.617	0.656	0.675	0.649	−0.206 ***
Seed7mer	0.578	0.623	0.626	0.609	−0.246 ***
Seed6mer + bulge	0.608	0.593	0.611	0.604	−0.251 ***
TargetNet [2]	0.575	0.533	0.572	0.560	−0.295 ***
Seed8mer	0.523	0.524	0.536	0.528	−0.327 ***

Bold indicates the models proposed in this work (DeepExoMir v19 and DeepExoMir-Lite). *** paired bootstrap p<0.001 vs. v19 on all three test sets. ^†^ DeepExoMir-Lite vs. v19 is operationally equivalent: Hejret p=0.075 n.s., Klimentová p=0.64 n.s., Manakov p<0.001 with effect |Δ|=0.004 (negligible; see the retrain-from-scratch ablation in Section 2.3). v19 was pre-specified before the ablation study as the reference model benchmarked against all eight baselines; DeepExoMir-Lite emerged from retrain-from-scratch ablation as a simplified variant with ≈35% fewer trainable parameters and no ViennaRNA dependency.

Our contribution is therefore methodological: we present a *systematically ablated* reference implementation and a dual-probe protocol that can be applied to future miRNA target prediction methods. The protocol reveals that within the design space we explored, hand-crafted conservation and structural features are approximately redundant with a frozen RNA language model’s implicit priors; a simpler architecture that omits the structural module would match v19’s benchmark performance. We retain v19 as the benchmarked reference model because it is the model evaluated against the eight miRBench baselines, but we emphasize that the ablation findings directly inform recommendations for future work.

**Table 2:** Development milestones across model versions (validation ROC-AUC only; descriptive development history). v19 is the *pre-specified* reference model for the Table 1 benchmark; v22 is an extended exploratory variant. Test-set performance on the three miRBench hold-out sets is reported separately in Table 1 (AU-PRC) to keep held-out-test evaluation compartmentalized from development-time model selection. This table is a descriptive development history, not a formal model-selection experiment.

Version	Key Change	Features	Val ROC-AUC
v1	Baseline MLP	0	0.593
v8	HybridEncoder + MoE + MultiTask	8	0.829
v10+	6-ch BP matrix + Mixup	12	0.833
v14-2L	+PhyloP conservation	31	0.850
v16a	Feature pruning (−8 noise)	23	0.850
v18	+miRNA secondary structure	33	0.850
**v19 (pre-specified reference)**	+InteractionPool + 4×CrossAttn	33	**0.852**
v22	+DuplexGAT (exploratory)	33	0.851

Bold marks the pre-specified reference model (v19).

**Table 3:** Retrain-from-scratch ablation results. Each ablation variant is trained from scratch with one signal source zeroed at every sample. Per-test-set AU-PRC and paired sample-level bootstrap versus v19 main are reported. Positive Δ indicates v19 main outperforms the ablation; negative Δ indicates the ablation outperforms v19 main. *** p<0.001, ** p<0.01, n.s. otherwise.

Ablation	Test Set	Ablated AU-PRC	Δ (Main−Abl.)	95% CI	*p*
v19_noRNALM	Hejret	0.814	+0.035 **	[+0.014, +0.056]	0.0014
v19_noRNALM	Klimentova	0.843	+0.025 ***	[+0.011, +0.039]	0.0006
v19_noRNALM	Manakov	0.822	+0.026 ***	[+0.025, +0.027]	<0.001
v19_noConservation	Hejret	0.842	+0.007 n.s.	[−0.007, +0.021]	0.352
v19_noConservation	Klimentova	0.864	+0.004 n.s.	[−0.003, +0.011]	0.316
v19_noConservation	Manakov	0.845	+0.003 ***	[+0.003, +0.004]	<0.001
v19_noStructure	Hejret	0.866	−0.017 n.s.	[−0.037, +0.002]	0.075
v19_noStructure	Klimentova	0.871	−0.003 n.s.	[−0.014, +0.009]	0.640
v19_noStructure	Manakov	0.853	−0.004 ***	[−0.005, −0.004]	<0.001

## 2. Results

### 2.1. DeepExoMir Maintains Performance Where Existing Methods Collapse

We first quantified the gap between self-reported performance obtained under random-negative settings and performance under rigorous miRBench evaluation with CLIP-seq-validated negatives (Figure 2). Across published methods, AU-PRC on the bias-corrected miRBench datasets falls to 0.55–0.80, substantially below the high performance values reported in the original studies under less rigorous evaluation settings. To enable rigorous head-to-head comparison under identical conditions, we used the miRBench Python package v1.0.1 [4] to run eight retrained baselines (TargetScanCnn, miRNA_CNN_Hejret, TargetNet, InteractionAware, and four seed-match heuristics: Seed6mer, Seed6mer+bulge, Seed7mer, Seed8mer) on the same three held-out test datasets (Hejret-CLASH n=965, Klimentova-eCLIP n=954, Manakov-eCLIP n=327,129), collected per-sample probability scores, and applied paired sample-level bootstrap (Table 1). DeepExoMir achieves mean AU-PRC 0.855 and outperforms *every* baseline on *every* test set with paired two-sided p<0.001; point-estimate AU-PRC gains over baselines span +0.075 (vs. the strongest baseline, miRNA_CNN_Hejret, on its native Hejret test set) to +0.325 (vs. Seed8mer). For additional reference, Sammut et al. [4] report a best-case retrained CNN baseline reaching mean AU-PRC ∼0.82, over which DeepExoMir retains a ∼0.04 AU-PRC advantage.

**Statistical significance of the performance gap.** Paired sample-level bootstrap (10,000 resamples; 2000 for Manakov) on per-sample prediction scores showed DeepExoMir outperforming every baseline on every test set at p<0.001, with point-estimate AU-PRC gains from +0.075 (vs. miRNA_CNN on Hejret) to +0.344 (vs. Seed8mer on Klimentová). Multi-seed training (seeds 42, 123, 456; Section 2.5) confirms reproducibility (test-set AU-PRC SD ≈0.001). Full per-comparison statistics in Table 1.

### 2.2. Signal Dependence: Complementary During Development, RiNALMo-Dominant at Inference

To characterize how the trained v19 model uses each signal source, we performed two complementary ablation analyses: (i) a cumulative-addition study across our model-evolution series (v1→v19), which shows what each newly-introduced signal contributed to optimization during development; and (ii) an inference-time feature-masking ablation on the v19 checkpoint, which probes which inputs the final trained model actively depends on at prediction time (Figure 3). The two probes yield distinct, partially divergent conclusions: each added signal improved training-time optimization, but at the final operating point the RiNALMo embedding carries almost all of the performance. We therefore characterize the three signals as *complementary during development but not independently necessary at inference-time for this trained model*, and we avoid the stronger claim that all three signals are *independently necessary* at prediction time.

Cumulative addition (Figure 3B; development-time metric: ROC-AUC) shows how performance improved as each prior was added: RiNALMo embeddings produced the largest gain, followed by smaller incremental additions from structural and conservation features. Inference-time feature masking (Figure 3A; metric: AU-PRC, to match the miRBench benchmark) reveals an asymmetric dependence: removing RiNALMo reduces Hejret AU-PRC by 0.055 and Klimentová by 0.161, whereas zeroing conservation or structural features produces near-zero effects (−0.0002 to +0.051). Conservation and structural features appear to capture partially redundant information that improved optimization but are not independently required at inference. The retrain-from-scratch ablations in Section 2.3 extend this analysis to training-time necessity within this architecture.

Table 2 is presented as a *descriptive development history* rather than a formal model-selection experiment; the final reference comparison reported in this paper is the pre-specified v19 checkpoint evaluated against the eight miRBench retrained baselines in Table 1 plus the retrain-from-scratch ablations in Table 3.

Key patterns from this descriptive development history (Table 2): (i) pre-trained RiNALMo embeddings were associated with the largest val-ROC-AUC gain (≈+0.15 over the baseline encoder; see Figure 3B); (ii) adding PhyloP conservation features (v10+ → v14-2L) provided modest val-ROC-AUC gains; (iii) removing eight legacy pairing features (v14-2L → v16a) preserved val performance while reducing noise; (iv) architectural refinements (InteractionPool + 4×CrossAttn for v19; DuplexGAT for v22) produced small incremental val gains. Table 2 reports validation ROC-AUC only; test-set performance on the three miRBench hold-out sets is reported separately in Table 1 (AU-PRC) to keep held-out-test evaluation compartmentalized from development-time model selection. Because several architectural milestones were nevertheless evaluated on the miRBench test sets during development, the Table 1 benchmark should be interpreted as a *standardized comparison under identical miRBench evaluation conditions* rather than a fully untouched final test evaluation. Benchmark comparisons against prior methods are reported in AU-PRC (Table 1; Figure 2); within-family development progression is tracked in ROC-AUC (Table 2; Figure 3B).

### 2.3. Retrain-from-Scratch Ablations Confirm RiNALMo Dominance and Signal Redundancy

Figure 3A’s inference-time feature-masking probes a trained checkpoint’s operating-point dependence on each signal. A complementary probe—retraining the model from scratch with one signal zeroed at every sample during both training and evaluation—asks whether that signal is *operationally necessary within this architecture during optimization*. We implemented three such variants (v19_noRNALM: RiNALMo embeddings zeroed; v19_noConservation: five PhyloP positions zeroed; v19_noStructure: base-pairing matrix plus 28 non-PhyloP structural features zeroed), each trained from scratch with identical hyperparameters and seeds, then evaluated on the three miRBench test sets with paired sample-level bootstrap versus v19 main.

Retrain-from-scratch results (Table 3) converge on the same signal hierarchy as the inference-time probe: **RiNALMo is operationally necessary** (v19_noRNALM loses 0.025–0.035 AU-PRC on every test set, all p≤0.0014; mean drop 0.029, smaller than inference-time masking 0.055–0.161 because a retrained-without model partially compensates via structure+conservation+architecture); **PhyloP is redundant** (v19_noConservation: *p* = 0.32–0.35 n.s. on small-n sets, only +0.003 on Manakov reaching significance due to n=327,129); **structural features are approximately redundant** (v19_noStructure mean 0.863 is within 0.008 of v19 main, with no significant per-test-set advantage for v19 on Hejret or Klimentova; a simpler architecture omitting the structural module would match v19’s benchmark performance).

Qualitatively the two probes agree: RiNALMo is the essential signal; conservation and structure contribute at most marginally at the trained-model operating point. They differ in magnitude for RiNALMo (retrain −0.029 vs. inference-time masking −0.055 to −0.161). A trained model relies more heavily on its final operating point than a freshly trained model relies on any single signal during optimization; the gap between the two probes reflects this. Per-comparison statistics are in Supplementary Section S7 (Tables S3 and S4).

**Effect size vs. significance.** Because Manakov has n=327,129, AU-PRC differences as small as 0.003 reach p<0.001. We interpret |Δ| below 0.01 as operationally negligible unless directionally consistent across all three test sets—only v19_noRNALM meets that bar here.

We designate v19_noStructure **DeepExoMir-Lite** (mean test AU-PRC 0.863) and recommend it as a simpler production variant (Table 1 footnote; Discussion Section 3.2). v19 main is retained as the pre-specified reference for baseline comparisons and retrospective validation.

### 2.4. Feature-Level Permutation Importance (v14alt2L)

As a complementary diagnostic during model development, we performed permutation importance analysis on an earlier 31-feature variant (v14alt2L; 50,000 validation samples, 3 repeats) by shuffling each biological feature independently and measuring the AU-PRC drop on the held-out validation subset. AU-PRC was chosen here (rather than ROC-AUC used for the development progression in Table 2) because permutation importance is an inference-time-style diagnostic applied to a specific trained checkpoint, and AU-PRC is the inference-time metric used by miRBench and throughout our ablation analyses (Figure 3A). Using this diagnostic and normalizing over features with positive importance only (legacy pairing-statistics features were excluded because they contributed negligible or slightly negative importance), the five conservation/site-location features (PhyloP mean, max, seed-mean, site_in_3’UTR, site_in_cds) together accounted for approximately 52%, thermodynamic features (12 including ensemble MFE and duplex MFE) for approximately 42%, and ViennaRNA accessibility features (6) for approximately 6% (sum ≈ 100%). The latter observation directly motivated the pruned feature set used in v16a/v18/v19. Note that permutation importance probes feature-level information content on the trained v14alt2L model and differs conceptually from the signal-level inference-time ablation in Figure 3A: permutation injects out-of-distribution noise whereas zeroing sets features to values consistent with missing-data training examples. The two probes are therefore complementary, and we report both. Exact per-feature values are available in the project repository (see Data Availability Statement).

### 2.5. Multi-Seed Stability

To quantify run-to-run variability, we re-trained v19 from scratch with three additional random seeds (42, 123, 456) and compared against the original v19 checkpoint, giving four independent training runs in total. Across these four runs, test ROC-AUC was 0.831±0.001 (mean ± SD) and test AU-PRC was 0.850±0.001, demonstrating robust performance across initializations (Table 4).

### 2.6. Attention Patterns Provide an Interpretability Signal Consistent with Canonical miRNA Biology

**Figure 4:**
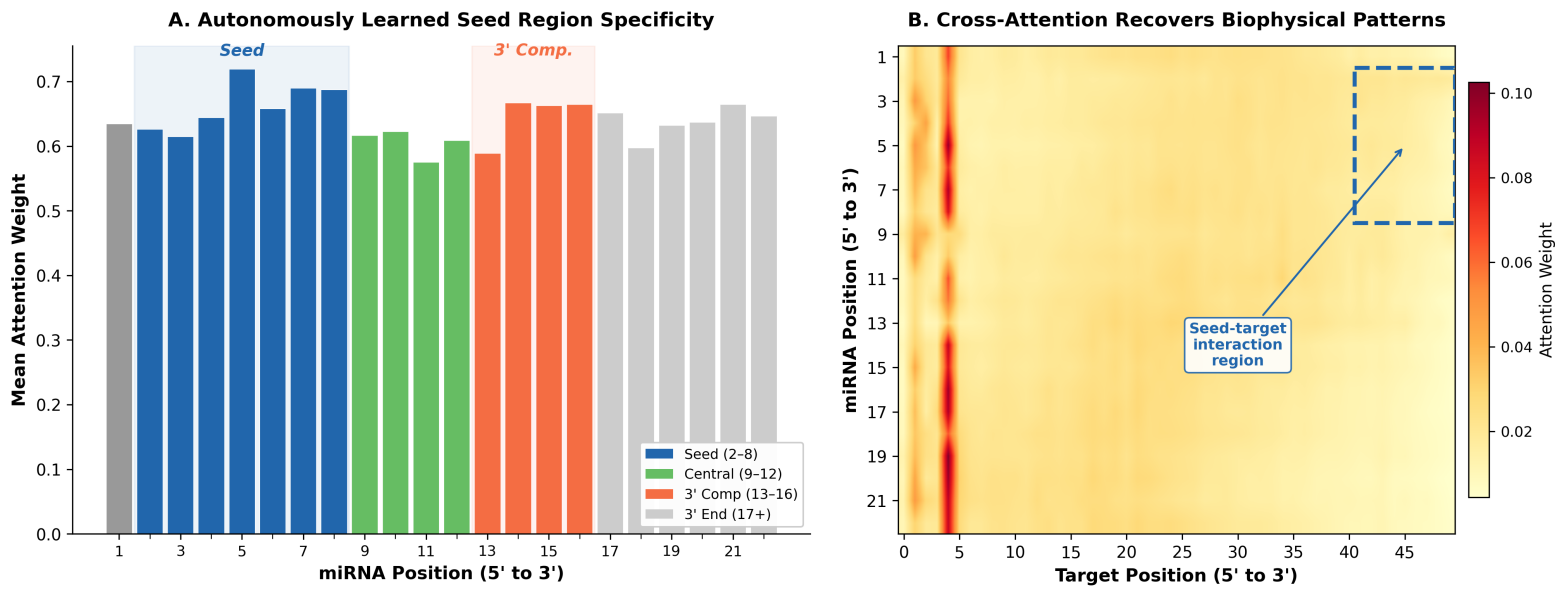
DeepExoMir’s attention patterns are consistent with canonical miRNA biology (interpretability signal, not mechanistic validation). (**A**) Mean cross-attention weight per miRNA position, averaged across 5000 positive test samples. Without explicit position supervision, the model concentrates attention on the canonical seed region (positions 2–8, blue). The 3’ compensatory region (positions 13–16, orange) exhibits mildly elevated attention. (**B**) Cross-attention heatmap showing that miRNA seed positions (rows 2–8) attend most strongly to the target 3’ end, qualitatively matching the expected geometry of miRNA-target duplex formation. Attention weights do not constitute mechanistic evidence—they indicate only that the trained model’s focus is consistent with known miRNA biology.

As a qualitative interpretability check—not a mechanistic validation—we examined whether DeepExoMir’s attention weights are consistent with known miRNA targeting biology. Attention weights do not, on their own, establish mechanism (as discussed in ref [26]); we use them here only to probe whether the trained model’s focus is *consistent with*, or *inconsistent with*, known miRNA-target interaction principles. We analyzed cross-attention weights across 5000 held-out positive samples (Figure 4). Without any explicit supervision on position identity, the first cross-attention layer exhibits elevated mean attention at miRNA positions 2–8 (peaking around positions 3–7; Figure 4A), corresponding to the canonical seed region responsible for Watson–Crick pairing with target mRNA [6]. The 3’ compensatory region (positions 13–16) also shows mildly elevated attention, consistent with the established role of supplementary 3’ pairing [7]. The cross-attention heatmap (Figure 4B) shows that miRNA seed positions attend most strongly to the target 3’ end, qualitatively consistent with the expected biophysical geometry of miRNA-target duplex formation. We stress that these observations only provide *interpretability support consistent with canonical miRNA biology* and do not establish that the model’s predictions are mediated by the same mechanisms observed experimentally.

### 2.7. Retrospective Validation on Experimentally Characterized Exosomal miRNAs

**Nature and scope.** All evidence in this section is *indirect*: we assess how DeepExoMir’s per-gene rankings recover literature-validated targets, canonical master regulators, and expected pathway annotations, with permutation-based FDR control. We do not perform direct functional validation (e.g., luciferase reporter assays); those experiments are explicit future work. These analyses support that DeepExoMir produces biologically sensible rankings that can prioritize candidates for wet-lab follow-up, not that every predicted target is a genuine causal target.

To assess generalization beyond miRBench, we ran DeepExoMir against nine exosomal miRNAs experimentally characterized for melanogenesis regulation in our companion study [20]: five novel miRNAs that reduce melanin production (*hsa-miR-6862-5p*, *hsa-miR-3622b-5p*, *hsa-miR-7847-3p*, *hsa-miR-6774-5p*, *hsa-miR-4685-5p*) and four literature-known miRNAs that enhance it (*hsa-miR-203a-3p*, *hsa-miR-126-3p*, *hsa-miR-139-5p*, *hsa-miR-15b-5p*).

**Figure 5:**
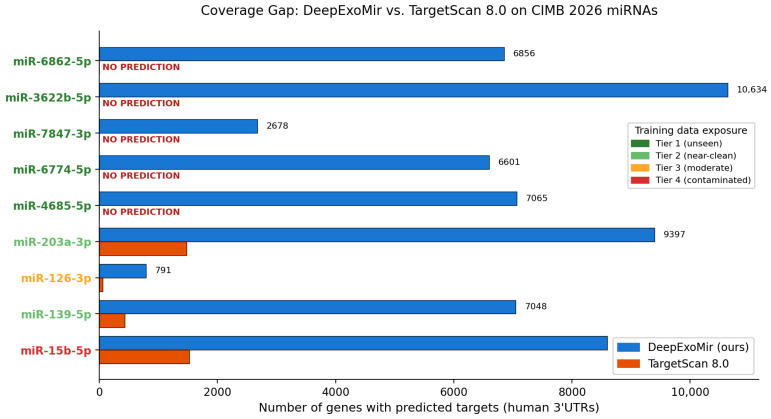
DeepExoMir provides candidate predictions for novel exosomal miRNAs that fall outside TargetScan 8.0’s database coverage. Y-axis labels are color-coded by training-data exposure tier under the v19 benchmark model: dark green = effectively unseen (Tier 1, *n* = 5), light green = near-clean (Tier 2, *n* = 2), orange = moderate exposure (Tier 3, *n* = 1), red = contaminated (Tier 4, *n* = 1; *hsa-miR-15b-5p*, for which a dedicated retrain sensitivity analysis is provided). TargetScan 8.0 provides no predictions for any Tier-1 miRNA (a consequence of its miRBase 22.1 cutoff); DeepExoMir generates 2678–10,634 ranked candidate target predictions per miRNA in that regime.

**Leakage disclosure.** Before making generalization claims, we verified training exposure via direct string matching of each miRNA identifier against miRBench’s 1.9M pairs (Figure 5): the five “reduce melanin” miRNAs occur 0–6 times (≤0.0003%, Tier 1, effectively unseen); *hsa-miR-203a-3p* and *hsa-miR-139-5p* appear 7 and 94 times (Tier 2, near-clean); *hsa-miR-126-3p* 2360 times (Tier 3); *hsa-miR-15b-5p* 51,586 times (Tier 4, 2.7% contaminated). Primary retrospective results use our v19 checkpoint with per-miRNA tier disclosed; for Tier 4, we additionally ran a dedicated retraining sensitivity analysis (v19_no15b checkpoint, retrained from scratch after removing every *hsa-miR-15b-5p*-containing row from the full dataset, 56,963 in total) to confirm the signal is not memorization.

**Prediction availability.** We ran DeepExoMir on 19,366 TargetScan 8.0 3’UTRs per miRNA, identifying canonical 6–8mer seed sites and scoring 117,875 candidate pairs in 485 s on a single RTX 5090. Because TargetScan 8.0 is built from miRBase 22.1, it provides zero predictions for the five Tier-1 novel miRNAs; DeepExoMir generates 2678–10,634 ranked candidates per miRNA in that regime (Figure 5). For the four miRNAs covered by both tools, DeepExoMir predicts 791–9397 genes versus TargetScan’s 60–1522. This difference reflects the two tools’ different design choices and should not be read as biological validation of one over the other; it is a practical statement that for novel miRNAs outside TargetScan’s database, DeepExoMir produces ranked candidates usable for downstream follow-up.

**Pipeline verification.** The retrospective pipeline computes 28 of 33 features from sequence alone; 5 PhyloP features are set to zero (precomputed vertebrate alignment not included) and site_in_3’UTR is 1.0 by construction. Rescoring the Hejret test set with identical code gives ROC-AUC 0.823 and AU-PRC 0.846 (versus 0.830 and 0.851 with the full pipeline), a <1% gap consistent with the minimal PhyloP contribution shown in Figure 3A.

**Figure 6:**
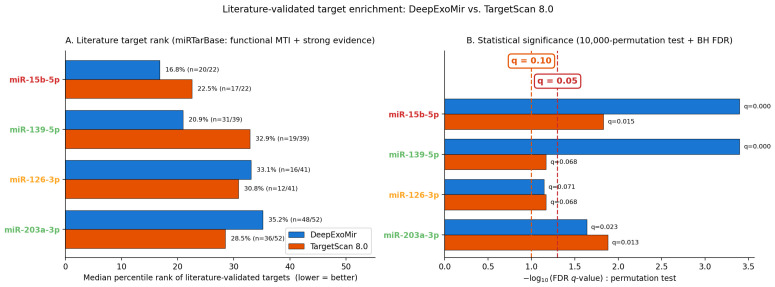
Literature-validated target recovery: DeepExoMir versus TargetScan 8.0. (**A**) Median percentile rank of experimentally verified miRNA-target interactions (miRTarBase functional MTI, strong evidence) among each tool’s predictions. Lower is better. Labels show percentile and fraction of literature targets found. (**B**) FDR-corrected permutation *q*-values (10,000 label shuffles, Benjamini–Hochberg). DeepExoMir reaches q<0.001 for *hsa-miR-15b-5p* and *hsa-miR-139-5p*. Y-axis labels color-coded by training-data exposure tier.

**Literature-validated target ranks.** We extracted 173 experimentally verified miRNA-target interactions from miRTarBase [27] (functional MTI, strong evidence: luciferase + Western blot + qRT-PCR) for the four literature-known miRNAs and tested whether the observed median rank is better than expected by chance via 10,000 label permutations with Benjamini–Hochberg FDR correction (Figure 6). Using the v19 benchmark checkpoint, DeepExoMir captures 31/39 *hsa-miR-139-5p* targets at median top-20.9% (*q* = 0.0003), 48/52 *hsa-miR-203a-3p* targets at top-35.2% (*q* = 0.023), 20/22 *hsa-miR-15b-5p* targets at top-16.8% (*q* = 0.0004), and 16/41 *hsa-miR-126-3p* targets at top-33.1% (*q* = 0.071). The v19_no15b sensitivity analysis recovers 20/22 *hsa-miR-15b-5p* targets (including BCL2, CCND1, CCNE1, CHEK1) at top-14.4% ($q<10^{-4}$), consistent with non-trivial ranking behavior beyond direct training exposure rather than simple memorization. TargetScan 8.0 captures fewer targets overall at comparable precision (*hsa-miR-139-5p* 19/39; *hsa-miR-15b-5p* 17/22 at top-22.5%). The five Tier-1 miRNAs have ≤1 miRTarBase entries each, consistent with their identification as newly discovered regulators.

**Figure 7:**
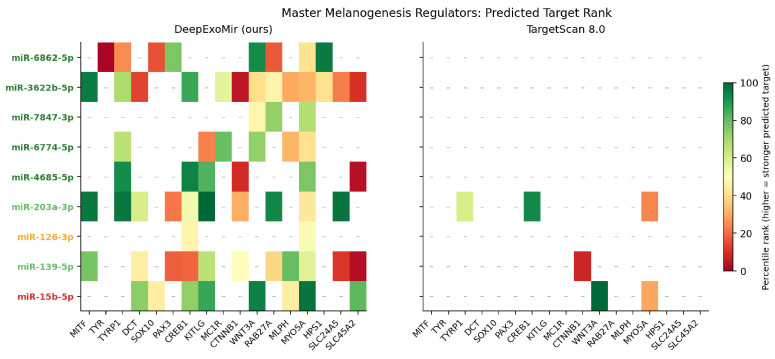
Percentile-score heatmap for 17 master melanogenesis regulators across nine miRNAs, comparing DeepExoMir (**left**) versus TargetScan 8.0 (**right**). Cell color encodes *percentile score* (defined here as 100−percentilerank, so that higher values = stronger predicted target; this convention is opposite to that used in Figure 6, where lower percentile rank = better, but matches the intuitive “brighter cell = stronger target” visual encoding). A dash indicates the gene is not among the tool’s predicted targets. DeepExoMir provides dense coverage across all miRNAs, whereas TargetScan is essentially silent for Tier-1 miRNAs.

**Master regulator recovery.** For *hsa-miR-203a-3p* (Tier 2, only 7 training rows out of 1.9 M), DeepExoMir places canonical melanogenesis regulators at the top of the 9397-gene ranking (Figure 7): KITLG rank 28 (**top 0.3%**), SLC24A5 top 2.9%, MITF top 3.7%, TYRP1 top 3.9%, LEF1 top 4.9%, RAB27A top 6.8%. TargetScan returns a different set (CREB1 rank 108, TYRP1 rank 590). The two tools appear to pick up different parts of the regulatory network: DeepExoMir leans toward biosynthetic enzymes and melanosome components, TargetScan toward transcriptional regulators.

**Figure 8:**
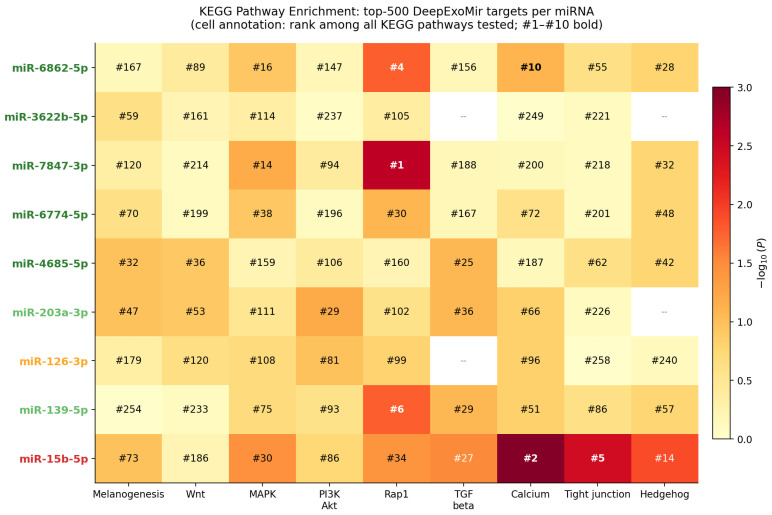
KEGG pathway enrichment (Enrichr) for the top-500 DeepExoMir predicted targets per miRNA. Cell color encodes $-\log_{10}\left(P\right)$; the “#” annotation in each cell is the pathway’s rank among all KEGG pathways tested for that miRNA (e.g., “#4” means 4th), with ranks #1–#10 in bold. Y-axis labels are color-coded by leakage tier. A dash indicates the pathway was not detected for the miRNA.

**Pathway enrichment.** KEGG pathway enrichment on the top 500 predictions per miRNA (Enrichr; BH-FDR across 90 tests; Figure 8) yields three significant hits (q<0.1): *hsa-miR-7847-3p* (Tier 1, zero training rows) enriches the **Rap1 signaling pathway** at rank 1 (*q* = 0.099, 13/210 genes); *hsa-miR-15b-5p* enriches Calcium signaling at rank 2 (*q* = 0.091) and Tight junction at rank 5 (*q* = 0.099). Rap1 and Calcium are canonical upstream regulators of melanogenesis, so recovering Rap1 for a genuinely out-of-distribution miRNA is an *indirect* but biologically meaningful signal. For *hsa-miR-126-3p*, the **Melanoma pathway** (KEGG hsa05218) ranks fifth with *P* = 0.017 (marginal after FDR).

**Figure 9:**
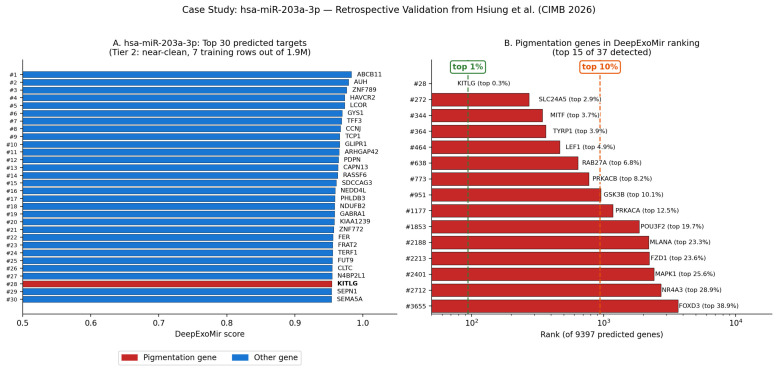
Detailed case study of *hsa-miR-203a-3p* (Tier 2: 7 training rows in 1.9 M) [20]. (**A**) Top 30 predicted targets. Red bars indicate pigmentation genes; blue bars indicate other genes. KITLG (rank 28, top 0.3%) enters the top-30 view directly. (**B**) The 15 highest-ranked pigmentation genes among DeepExoMir’s 9397 predictions. Vertical dashed lines denote top 1% (green) and top 10% (orange) of the full ranking. Canonical melanogenesis regulators cluster in the top 10%: KITLG (top 0.3%), SLC24A5 (2.9%), MITF (3.7%), TYRP1 (3.9%), LEF1 (4.9%).

**Case study:** ***hsa-miR-203a-3p***. Figure 9 examines this near-clean Tier-2 miRNA in more detail. Panel A shows the top 30 predicted targets; most are non-pigmentation genes (blue), as expected for any miRNA that regulates beyond a single phenotype. Panel B shows that nine pigmentation genes (TYRP1, SLC24A5, KITLG, LEF1, PRKACB, plus four others) sit inside DeepExoMir’s top 5%, even though the broader target spectrum is mostly non-pigmentation.

Together these analyses (a) are consistent with non-trivial ranking behavior on Tier-1/2 miRNAs effectively unseen in training, (b) show DeepExoMir provides predictions where TargetScan is silent, and (c) recover canonical melanogenesis regulators at meaningful percentile ranks. They do not constitute direct target-level biological validation. Three control analyses (miRNA-permutation null, random-score negative control, random gene-set control) confirm the signal is not a gene-universe artifact; full details are in Supplementary Section S2. The random gene-set control returns a null result; this is not evidence against the method but reflects the coarseness of a 57-gene reference set as an enrichment endpoint.

### 2.8. Score Calibration and Prioritization Thresholds

**Figure 10:**
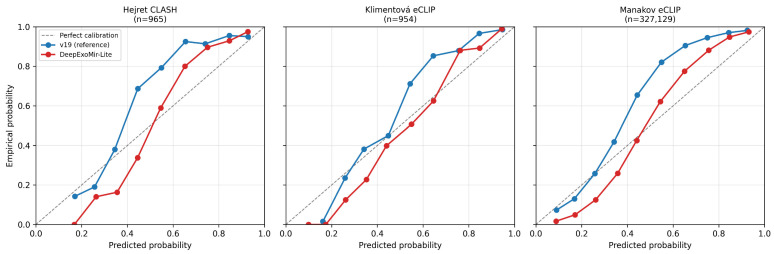
Calibration of DeepExoMir output scores across the three miRBench test sets. Reliability diagrams (10 bins) compare predicted probability against empirical positive rate; the dashed diagonal indicates perfect calibration. Both v19 main (blue) and DeepExoMir-Lite (red) show moderate calibration with ECE 0.07–0.12 and Brier 0.16–0.19. Lite achieves lower Maximum Calibration Error (MCE 0.14–0.19) than v19 (MCE 0.21–0.27) on all three test sets, indicating slightly tighter worst-case calibration. Scores are best interpreted as ranking signals; percentile-based thresholds are recommended for cross-miRNA prioritization.

Beyond aggregate AU-PRC, we evaluated whether DeepExoMir output scores are well-calibrated as probabilities. Reliability diagrams (Figure 10) compare predicted probabilities against empirical positive rates across ten equally spaced bins on the three miRBench test sets, for both v19 main and DeepExoMir-Lite. Expected Calibration Error (ECE) ranges from 0.07 (Klimentová v19) to 0.12 (Hejret), and Brier scores from 0.16 to 0.19. Lite shows slightly lower Maximum Calibration Error (MCE) than v19 main on all three test sets (0.14–0.19 vs. 0.21–0.27). Both models tend to under-confidence at lower probability ranges, particularly on the larger Manakov set. We therefore recommend interpreting DeepExoMir scores as *ranking signals rather than absolute probabilities*: percentile-based thresholds (top-1%, top-5%, top-10%) provide more stable prioritization across miRNAs than absolute score cutoffs. This caveat is consistent with the field’s broader observation that CLIP-seq-supervised models reflect the labeling-positive distribution of the training data and do not directly estimate functional repression probability.

## 3. Discussion

### 3.1. Signal Diversity Within the Explored Design Space

Within the design space we explored, combining a frozen RNA language model with auxiliary biological priors transfers better to CLIP-seq-validated negatives than scaling architectural depth within any single signal. This is not a universal law: our claim covers a specific architecture family (v19 plus published CNN, transformer, and attention-based miRNA target predictors evaluated on miRBench), is not a controlled comparison under matched training budgets, and is restricted to human miRNA-target interaction prediction. Other signal combinations or architectures may dominate elsewhere.

Two practical implications follow. The first is that single-signal architectures break down under realistic evaluation. Methods relying primarily on sequence-level patterns, regardless of model class (CNN, transformer, attention-based), collapsed from self-reported values above 0.90 to AU-PRC 0.55–0.80 on miRBench’s CLIP-seq bias-corrected datasets (Figure 2). The collapse is not about deep learning as a paradigm. It is about random-negative training letting models exploit low-level sequence statistics that do not transfer. The problem is the *signal* the architecture was asked to learn, not the architecture.

The second is that within our hybrid, the RiNALMo embedding is the dominant signal. Inference-time feature-masking (Figure 3A) reduces Hejret AU-PRC by 0.055 and Klimentová AU-PRC by 0.161 when RiNALMo is zeroed, while zeroing PhyloP or the entire structural-feature/base-pairing-matrix block produces changes within noise. Retrain-from-scratch (Section 2.3) returns the same ranking: v19_noRNALM drops mean AU-PRC by 0.029 (p<0.001 on Klimentová and Manakov), while v19_noConservation and v19_noStructure stay within 0.008 of v19 main. The conclusion: RiNALMo carries most of the predictive signal, and explicit hand-crafted conservation or structural features are largely redundant with the implicit priors RiNALMo absorbed from pre-training on 36 M non-coding RNA sequences [17]. This fits the growing literature on frozen foundation models as feature extractors for biological downstream tasks [28,29,30,31]. Our brief fine-tuning experiments produced no performance gain while roughly quadrupling training time. PCA reduction (1280 → 256, retaining 88.7% variance) keeps deployment cheap. These observations are properties of this trained model on this benchmark, not a universal recipe.

### 3.2. Dual-Probe Convergence and DeepExoMir-Lite

The retrain-from-scratch ablations (Section 2.3) do more than confirm the inference-time masking results: they rule out the hypothesis that hand-crafted conservation and structural features are *operationally necessary within this architecture during optimization*. v19_noConservation and v19_noStructure are trained from scratch with the corresponding signals zeroed at every sample, yet both converge to mean test AU-PRC within 0.008 of v19 main.

**DeepExoMir-Lite.** We designate v19_noStructure (RiNALMo + PhyloP + v19 backbone, no base-pairing CNN, no ViennaRNA descriptors) as **DeepExoMir-Lite** and recommend it for production deployment (see Table 1 footnote; full per-test-set statistics in Table 3).

We also observe an internally inconsistent ranking among the three retrain variants: v19_noStructure had the *lowest* best-epoch val ROC-AUC (0.824 vs. 0.841/0.854 for v19_noRNALM/v19_noConservation) yet achieved the *highest* mean test AU-PRC (0.863). Because val ROC-AUC and test AU-PRC quantify different aspects of performance we do not treat this as a direct contradiction, but it cautions against val-ROC-AUC-only architecture selection—a methodological caveat applicable to the miRNA target prediction field more broadly. Two points temper this caveat in practice. First, the three retrain variants differ on test AU-PRC by at most 0.008, which is within the multi-seed run-to-run standard deviation (about 0.001 to 0.008 AU-PRC; Section 2.5); on test performance they are effectively tied, so no clearly superior model was at risk of being discarded. Second, for future selection we recommend monitoring the metric that matches the operational objective: because miRBench scores ranking under class imbalance, validation average precision (AU-PRC) is a more faithful selection criterion than validation ROC-AUC, and matching the positive-to-negative ratio and miRNA-frequency distribution of the validation split to the test regime further reduces the divergence we observe. Where the intended use is candidate prioritization, top-*k* precision or partial AU-PRC in the high-precision region is more closely aligned still.

### 3.3. Practical Guidance and Generalisability

Three practical points follow from the ablation analysis. First, on feature design: many established miRNA target predictors lean on cross-species conservation, exemplified by the context scoring of TargetScan [15], and on hand-crafted structural or accessibility descriptors [19,32]. Our dual-probe ablation indicates that, once a frozen RNA language model is present, these signals are largely redundant: removing PhyloP conservation changes mean test AU-PRC by less than 0.004 and removing the structural module raises it by 0.008, against the 0.029 cost of removing RiNALMo. Conservation and explicit structure can therefore be treated as optional rather than core in future designs. Second, on attribution method: a feature’s importance should be probed with both an inference-time mask (the operating-point dependence of the trained model) and a retrain-from-scratch ablation (training-time necessity) before it is judged dispensable, because the two can disagree, as they do here for RiNALMo. Third, the leakage-tier protocol is benchmark-agnostic: for any supervised target-prediction benchmark one can exact-match each query entity against the training partition, bin the exposure counts by order of magnitude, run a retrain-without check for any tier whose exposure could drive memorization, and report the tier alongside each performance number. For the panel here we set the effectively-unseen Tier 1 conservatively at the maximum exposure observed among the de-novo miRNAs (six rows), so that low single-digit cases fall in the next tier rather than being claimed as unseen. We make no claim of clean out-of-distribution generalization for Tier 2–3 miRNAs; we disclose their exposure and read the rankings accordingly, while the control analyses of Supplementary Section S2 apply at every tier.

The retrospective pipeline itself is mechanism-agnostic: it scores candidate sites, ranks them per gene, and tests enrichment against a curated reference set. Applying it to inflammatory or oncogenic contexts requires only swapping the reference gene set and, ideally, adding a tissue- or context-specific expression filter so that lowly expressed genes are not ranked as candidates; the melanogenesis case illustrates the workflow rather than bounding it. Operationally, the output is best used as a ranked shortlist: take the top percentile of candidates for a miRNA of interest, intersect them with a context-specific gene set, and carry the survivors into functional validation (luciferase reporter or CRISPR perturbation) or into the design of a targeted follow-up CLIP-seq experiment. Because the scores are calibrated as rankings rather than absolute probabilities (Section 2.8), percentile thresholds rather than fixed cutoffs should drive the shortlist, and this holds equally outside the pigmentation setting. Readers seeking the experimental characterization of these miRNAs (extracellular-vesicle isolation, melanin assays, and transfection validation) should consult the companion paper [20]; the present work covers the computational framework, benchmarking, and leakage-tier disclosure.

### 3.4. Limitations

Key limitations: (i) Evaluation is limited to human miRNA-target interactions; cross-species transfer is future work. The frozen RiNALMo backbone was pre-trained on a taxonomically broad non-coding-RNA corpus that includes plant, viral, and microbial sequences, so its embeddings plausibly already encode features relevant beyond human RNA. The species-specific component of DeepExoMir is therefore the trained classifier head rather than the representation: that head learned the seed-centric, 3’UTR-biased targeting rules of animal miRNAs from miRBench. We expect this to make virally encoded miRNAs, which use the host seed-based machinery, the more tractable cross-species case, whereas plant miRNAs, which bind their targets with near-perfect complementarity and often within coding regions, would require the head to relearn the targeting rule itself rather than adapt to it. A direct test is to re-train only the classifier head on species-specific degradome or CLIP-seq data, with the vertebrate PhyloP channel disabled as in our retrospective pipeline; we leave this to future work. (ii) Attention analysis is interpretive, not a substitute for perturbation experiments [26]. (iii) The retrospective scoring pipeline omits five PhyloP features (BigWig lookups not feasible for de novo windows); Hejret cross-validation bounds the impact at <1% ROC-AUC. (iv) Direct functional validation (luciferase, transfection-CLIP-seq) is outside scope; phenotypic effects were independently verified in [20]. (v) The three retrain-from-scratch ablation variants show an internally inconsistent val/test ranking (v19_noStructure had the lowest val ROC-AUC yet the highest test AU-PRC), cautioning against val-ROC-AUC-only architecture selection even though the two metrics are not directly comparable. (vi) The single contaminated miRNA (Tier 4, *hsa-miR-15b-5p*) was addressed by the v19_no15b retrain sensitivity analysis ($q<10^{-4}$). (vii) All three test sets are curated by miRBench; leave-one-study-out cross-validation and evaluation on CLIP-seq cohorts outside this ecosystem are explicit future work.

## 4. Materials and Methods

### 4.1. Datasets and Preprocessing

#### 4.1.1. Training Data

All training, validation, and test data were sourced from the miRBench standardized benchmark suite [4], which provides experimentally validated miRNA-target interaction datasets from AGO2 crosslinking experiments with controlled negative sampling. Three complementary datasets were combined: AGO2 eCLIP from Manakov et al. [33] (1,906,909 training pairs), AGO2 CLASH from Hejret et al. (5925 training pairs), and AGO2 eCLIP from Klimentova et al. (616 training pairs). The miRBench pipeline employs gene-level splitting to prevent data leakage, ensuring all pairs involving a given target gene are assigned entirely to one partition. After removing 45,863 contradictory pairs (identical miRNA-target tuples with conflicting labels), the final dataset comprised 2,761,229 samples split into training (1,913,450, 69.3%), validation (339,894, 12.3%), and test (507,885, 18.4%) sets.

#### 4.1.2. External Evaluation Benchmark

For comparison with published baselines, we evaluated on the three miRBench held-out test sets: Hejret-AGO2 CLASH (965 samples), Klimentova-AGO2 eCLIP (954 samples), and Manakov-AGO2 eCLIP (327,129 samples). These test sets use a balanced 1:1 positive-to-negative ratio with experimentally validated CLIP-seq negatives.

#### 4.1.3. Scope of Benchmark Claims

Our benchmarking is deliberately restricted to human miRNA-target interactions as represented in miRBench’s AGO2-centered CLIP-seq evaluation ecosystem. All training and test data derive from human samples; all negative examples originate from the same miRBench ecosystem; and the CLIP-seq technology itself carries known biases (cross-linking efficiency, AGO2 capture specificity, sequencing artifacts). Consequently, our performance claims apply to: *(i)* human miRNA-target prediction, *(ii)* under miRBench’s CLIP-seq-validated negative-sampling convention, *(iii)* against the specific baselines re-evaluated or retrained on the same benchmark. Generalization beyond these conditions—to non-human species, non-AGO2 Argonaute family members, or negative-sampling schemes outside miRBench’s conventions—is an open question that we explicitly do not claim to resolve here.

### 4.2. Sequence Embeddings

We employed the pre-trained RiNALMo-giga model [17], a 650M-parameter RNA language model. Per-token embeddings (dimension 1280) were extracted for all sequences. PCA dimensionality reduction from 1280 to 256 dimensions, retaining 88.7% of variance, reduced storage from 151 GB to 31 GB. Both per-token and mean-pooled embeddings were pre-computed and cached.

### 4.3. Model Architecture

DeepExoMir (Figure 1) integrates three signal streams: (1) PCA-reduced RiNALMo embeddings processed by an 8-layer hybrid encoder alternating BiConvGate (depthwise separable convolution + SwiGLU [34,35]) and 4 cross-attention layers; (2) explicit duplex structure via a 6-channel base-pairing CNN over the 30×50 alignment matrix; and (3) 33 pre-computed biological features spanning thermodynamic, ViennaRNA [19,36], PhyloP conservation [18], and miRNA-secondary-structure descriptors [32,37]. A multi-head interaction-pooling layer (4 heads) produces a 512-d joint representation, which is concatenated with biological features and routed through a 4-expert top-2 Mixture-of-Experts classifier [38] with Platt scaling. Auxiliary multi-task heads (seed binding strength, duplex MFE regression, site position) provide additional training signal. Each head predicts a biophysically interpretable quantity (seed-region complementarity, thermodynamic duplex stability, and 3’UTR binding-site location), but the heads were included to regularize the shared representation rather than as standalone predictors and were not benchmarked as separate outputs. An extended variant (v22) replaces the base-pairing CNN with a duplex graph attention network (DuplexGAT) [39] using 80 nucleotide nodes and GATv2Conv layers [40,41,42]; v22 is exploratory and is reported alongside, not ahead of, v19. Full layer-by-layer specifications, hyperparameters, and ablation details are provided in Supplementary Section S3.

### 4.4. Training Procedure

Models were trained for up to 60 epochs with early stopping (patience 20, monitoring validation ROC-AUC). We used AdamW with cosine scheduling (peak LR 1e-4, 1000-step warmup), gradient accumulation over 8 micro-batches (effective batch size 2048), and FP16 mixed precision. Focal loss (gamma = 1.0, alpha = 0.5) with label smoothing (0.05) addressed class imbalance. Embedding-level Mixup (alpha = 0.2, *p* = 0.5) and sequence-level structural perturbation augmentation was applied. All experiments used a single NVIDIA RTX 5090 GPU (32 GB).

### 4.5. Computational Cost

Training and inference cost figures for the main v19 model are summarized in Table 5.

**Deployment profile.** Scoring with the v19 head is light: roughly 4600 (miRNA, site) pairs per second on a single RTX 5090, so a whole-transcriptome screen for one miRNA (about 19,000 3’UTRs, about 13,000 candidate sites after seed enumeration) completes in seconds, and a panel of a few hundred miRNAs in minutes. The 26.4M-parameter classifier is light; the memory and compute cost concentrates in the upstream RiNALMo-giga (650 M-parameter) embedding step, which in our runs occupies essentially the full 32 GB of an RTX 5090. We therefore recommend computing embeddings once on a GPU and caching them. Deployment does not require the full training-set embedding cache: only the query sequences’ embeddings are needed, and these can be computed on demand. The model itself trains and runs within roughly 12 GB at batch size 256, so the classifier is not the limiting factor on common hardware; the practical requirement is GPU memory sufficient for the RiNALMo embedding pass, which can be lowered by reducing the embedding batch size. Because the classifier head is small, scoring from cached embeddings is expected to be tractable on CPU for moderately sized jobs, although we have not formally profiled CPU-only inference; whole-transcriptome embedding generation should use a GPU.

### 4.6. Retrospective Validation Methodology

We applied the trained model to nine experimentally characterized exosomal miRNAs from our companion study [20]: five novel “reduce melanin” miRNAs (*hsa-miR-6862-5p*, *-3622b-5p*, *-7847-3p*, *-6774-5p*, *-4685-5p*) and four literature-known “enhance melanin” miRNAs (*hsa-miR-203a-3p*, *-126-3p*, *-139-5p*, *-15b-5p*). Per-miRNA training-row counts (from direct string matching against the 1.9M-pair miRBench training set) were used to classify miRNAs into four leakage tiers (clean/near-clean/moderate/contaminated). Transcriptome-scale scoring used 19,366 human 3’UTR sequences from TargetScan 8.0. Canonical 6–8mer seed sites were enumerated, and the **v19 main checkpoint** scored 117,875 candidate (miRNA, site) pairs for all nine miRNAs in 485 sec on an RTX 5090. For *hsa-miR-15b-5p* (the single Tier-4 contaminated miRNA) we additionally repeated scoring with the **v19_no15b sensitivity checkpoint**, retrained from scratch after removing all 56,963 *hsa-miR-15b-5p*-containing rows from the full dataset (training, validation, and test; 51,586 of these lie in the training split, the 2.7% exposure used for tier classification). Pipeline correctness was verified by re-scoring the Hejret CLIP-seq test set, obtaining ROC-AUC 0.823/AU-PRC 0.846 (vs. 0.830/0.851 with the full pipeline, a <1% ROC-AUC gap). KEGG pathway enrichment used Enrichr (KEGG_2021_Human via gseapy). Comparison baseline: TargetScan 8.0 default predictions, aggregated per gene by minimum context++ score. Complete protocol details, including per-tier exposure counts and pipeline-verification numerical comparison, are in Supplementary Section S4. We used these nine miRNAs because their melanin phenotypes were experimentally characterized in the companion study [20], which provides an independent biological anchor for retrospective scoring. The framework is not limited to them: it produces ranked predictions for any miRNA, including further candidates in our own pipeline that currently lack an independent experimental or literature anchor. We restricted the validation reported here to the literature- and experimentally-supported panel to avoid the circularity of treating the model’s own predictions as ground truth; experimental validation of the additional predicted miRNAs is ongoing and will be reported separately.

### 4.7. Reproducibility and Implementation Details

To ensure full reproducibility of our machine learning pipeline, all experiments were conducted with fixed random seeds (42, 123, 456 for stability testing) across data splitting, model initialization, and batch sampling. The framework is implemented in PyTorch 2.1.0 and PyTorch Geometric 2.4.0. Training was performed on a single NVIDIA RTX 5090 GPU (32 GB VRAM) running Ubuntu 22.04 with CUDA 12.1. The complete source code, including preprocessing scripts, model definitions, training loops, and retrospective validation pipelines, is open-sourced under the MIT license. The exact code and weights behind every reported number are archived under tagged releases (GitHub tag v1.1.0; Zenodo concept DOI 10.5281/zenodo.19216306). We do not run a continuous-integration service; reproducibility is instead enforced by pinned pytest smoke tests with fixed reference outputs and a CPU-only Dockerfile, which together regenerate the retrain-from-scratch ablation comparison (Table 3) and the calibration analysis from those fixed outputs.

## 5. Conclusions

DeepExoMir v19 reaches mean AU-PRC 0.855 on three independent miRBench test datasets and beats all eight retrained miRBench baselines at paired two-sided p<0.001. The dual-probe ablation protocol returns one consistent ranking. RiNALMo is the dominant and operationally necessary signal (retrain-from-scratch mean drop 0.029 AU-PRC, p<0.001 on two of three test sets). PhyloP conservation and explicit structural features are largely redundant with RiNALMo’s implicit priors (retrain-from-scratch deltas −0.004 and +0.008). We therefore release **DeepExoMir-Lite** (RiNALMo + PhyloP + v19 backbone, no base-pairing CNN, no ViennaRNA-dependent features) as a simpler production variant with mean test AU-PRC 0.863 and roughly 35% fewer parameters. v19 main is kept as the reference implementation against which all baseline comparisons and retrospective-validation results were generated. Attention analysis provides an interpretability signal consistent with canonical seed-region biology, not mechanistic validation.

Retrospective validation on nine exosomal miRNAs with four-tier leakage disclosure provides *indirect* evidence of practical applicability: DeepExoMir generates candidate predictions where TargetScan 8.0 does not, prioritizes canonical melanogenesis regulators, and recovers 20/22 literature targets at top-14.4% for the contaminated miRNA even after excluding all its training rows (FDR-$q<10^{-4}$). DeepExoMir is positioned as a *candidate-prioritization framework for downstream experimental validation*. Future work should include direct functional validation, leave-one-study-out cross-validation on external AGO2-CLIP cohorts outside the miRBench ecosystem, dual-probe extension to other architectures, and cross-species generalization.

DeepExoMir is freely available at https://github.com/linwenh09/DeepExoMir (accessed on 2 July 2026) and archived at Zenodo (https://doi.org/10.5281/zenodo.19216306).

## Figures and Tables

**Table 4:** Multi-seed evaluation (v19 architecture).

Seed	Val ROC-AUC	Test ROC-AUC	Test AU-PRC
Original	0.852	0.832	0.852
42	0.850	0.830	0.850
123	0.852	0.830	0.850
456	0.851	0.830	0.849
Mean ± SD	0.851 ± 0.001	0.831 ± 0.001	0.850 ± 0.001

**Table 5:** Computational requirements for DeepExoMir.

Component	Metric	Value
Model parameters	v19/v22	26.4 M/27.9 M
Embedding pre-computation	Time (1.9 M pairs)	~8 h
Embedding cache	Storage (PCA-256)	31 GB
Training time	v19 (55 epochs)	~14 h
Training time	v22 (48 epochs)	~21 h
Training throughput	Samples/sec	~37 (v19)/~25 (v22)
Per-epoch time	v19/v22	16 min/26 min
Inference time	508K test samples	~110 s
Inference throughput	Samples/sec	~4600
GPU memory	Training (batch = 256)	~12 GB
Checkpoint size	v19/v22	106 MB/112 MB

## Data Availability

All resources are deposited at Zenodo (https://doi.org/10.5281/zenodo.19216306) and mirrored at https://github.com/linwenh09/DeepExoMir (accessed on 2 July 2026) under the MIT license. The deposit contains the v19 and DeepExoMir-Lite checkpoints, the two retrain-from-scratch ablation checkpoints (v19_noConservation, v19_noRNALM), per-sample probability score files for all eight miRBench baselines and our four ablations on each of the three CLIP-seq test sets, the dual-probe ablation pipeline, the calibration analysis, the quickstart notebook (notebooks/01_quickstart.ipynb), and a CPU-only Dockerfile that reproduces the Table 3 retrain-from-scratch ablation comparison and the Figure 10 calibration analysis end-to-end in approximately three minutes through pytest tests/test_reproduce_table3.py tests/test_calibration.py. The miRBench benchmark datasets are obtainable through the miRBench Python package [4]. Detailed step-by-step reproducibility instructions are provided in REPRODUCIBILITY.md at the repository root.

## Supplementary Materials

The following supporting information can be downloaded at: https://www.mdpi.com/article/10.3390/ijms27146184/s1.

## Abbreviations

The following abbreviations are used in this manuscript:

miRNA	microRNA
RISC	RNA-induced silencing complex
UTR	untranslated region
PCA	principal component analysis
GATv2	Graph Attention Network version 2
MoE	Mixture-of-Experts
CNN	convolutional neural network
MLP	multilayer perceptron
MFE	minimum free energy
AU-PRC	area under the precision-recall curve
ROC-AUC	area under the receiver operating characteristic curve
EV	extracellular vesicle

## References

[1] Wen M., Cong P., Zhang Z., Lu H., Li T. (2018). DeepMirTar: A deep-learning approach for predicting human miRNA targets. Bioinformatics.

[2] Min S., Lee B., Yoon S. (2022). TargetNet: Functional microRNA target prediction with deep neural networks. Bioinformatics.

[3] Klimentová E., Hejret V., Krčmář J., Grešová K., Giassa I.-C., Alexiou P. (2022). miRBind: A Deep Learning Method for miRNA Binding Classification. Genes.

[4] Sammut S., Gresova K., Tzimotoudis D., Marsalkova E., Cechak D., Alexiou P. (2025). miRBench: Novel benchmark datasets for microRNA binding site prediction that mitigate against prevalent microRNA frequency class bias. Bioinformatics.

[5] Bartel D.P. (2018). Metazoan microRNAs. Cell.

[6] Bartel D.P. (2009). MicroRNAs: Target recognition and regulatory functions. Cell.

[7] Grimson A., Farh K.K., Johnston W.K., Garrett-Engele P., Lim L.P., Bartel D.P. (2007). MicroRNA targeting specificity in mammals: Determinants beyond seed pairing. Mol. Cell.

[8] Shin C., Nam J.W., Farh K.K., Chiang H.R., Shkumatava A., Bartel D.P. (2010). Expanding the microRNA targeting code: Functional sites with centered pairing. Mol. Cell.

[9] Chi S.W., Hannon G.J., Darnell R.B. (2012). An alternative mode of microRNA target recognition. Nat. Struct. Mol. Biol..

[10] Kozomara A., Birgaoanu M., Griffiths-Jones S. (2019). miRBase: From microRNA sequences to function. Nucleic Acids Res..

[11] Friedman R.C., Farh K.K., Burge C.B., Bartel D.P. (2009). Most mammalian mRNAs are conserved targets of microRNAs. Genome Res..

[12] Rupaimoole R., Slack F.J. (2017). MicroRNA therapeutics: Towards a new era for the management of cancer and other diseases. Nat. Rev. Drug Discov..

[13] Zheng X., Chen L., Li X., Zhang Y., Xu S., Huang X. (2020). Prediction of miRNA targets by learning from interaction sequences. PLoS ONE.

[14] Betel D., Koppal A., Agius P., Sander C., Leslie C. (2010). Comprehensive modeling of microRNA targets predicts functional non-conserved and non-canonical sites. Genome Biol..

[15] Agarwal V., Bell G.W., Nam J.W., Bartel D.P. (2015). Predicting effective microRNA target sites in mammalian mRNAs. eLife.

[16] Chen Y., Wang X. (2020). miRDB: An online database for prediction of functional microRNA targets. Nucleic Acids Res..

[17] Penić R.J., Vlašić T., Huber R.G., Wan Y., Šikić M. (2025). RiNALMo: General-purpose RNA language models can generalize well on structure prediction tasks. Nat. Commun..

[18] Pollard K.S., Hubisz M.J., Rosenbloom K.R., Siepel A. (2010). Detection of nonneutral substitution rates on mammalian phylogenies. Genome Res..

[19] Lorenz R., Bernhart S.H., Höner Zu Siederdissen C., Tafer H., Flamm C., Stadler P.F., Hofacker I.L. (2011). ViennaRNA package 2.0. Algorithms Mol. Biol..

[20] Hsiung C.-N., Lien W.-Y., Sieber M., Lin W.-H. (2026). Omic Profiling of Extracellular Vesicles from Two Cord-Related Sources Reveals Divergent Effects on Melanogenesis. Curr. Issues Mol. Biol..

[21] Valadi H., Ekström K., Bossios A., Sjöstrand M., Lee J.J., Lötvall J.O. (2007). Exosome-mediated transfer of mRNAs and microRNAs is a novel mechanism of genetic exchange between cells. Nat. Cell Biol..

[22] O’Brien K., Breyne K., Ughetto S., Laurent L.C., Breakefield X.W. (2020). RNA delivery by extracellular vesicles in mammalian cells and its applications. Nat. Rev. Mol. Cell Biol..

[23] McGeary S.E., Lin K.S., Shi C.Y., Pham T.M., Bisaria N., Kelley G.M., Bartel D.P. (2019). The biochemical basis of microRNA targeting efficacy. Science.

[24] Hejret V., Varadarajan N.M., Klimentova E., Gresova K., Giassa I.-C., Vanacova S., Alexiou P. (2023). Analysis of chimeric reads characterises the diverse targetome of AGO2-mediated regulation. Sci. Rep..

[25] Yang T.-H., Chen J.-C., Lee Y.-H., Lu S.-Y., Wu S.-H., Chang F.-Y., Huang Y.-C., Lee M.-H., Tseng Y.-Y., Wu W.-S. (2024). Identifying Human miRNA Target Sites via Learning the Interaction Patterns between miRNA and mRNA Segments. J. Chem. Inf. Model..

[26] Jain S., Wallace B.C. (2019). Attention is not explanation. NAACL-HLT.

[27] Huang H.Y., Lin Y.C., Li J., Huang K.Y., Shrestha S., Hong H.C., Tsai Y., Chen Y.H., Tsai H.D., Lee T.Y. (2022). miRTarBase update 2022: An informative resource for experimentally validated miRNA-target interactions. Nucleic Acids Res..

[28] Bommasani R., Hudson D.A., Adeli E., Altman R., Arora S., von Arx S., Bernstein M.S., Bohg J., Bosselut A., Brunskill E. (2021). On the opportunities and risks of foundation models. arXiv.

[29] Ji Y., Zhou Z., Liu H., Davuluri R.V. (2021). DNABERT: Pre-trained bidirectional encoder representations from transformers model for DNA-language in genome. Bioinformatics.

[30] Jumper J., Evans R., Pritzel A., Green T., Figurnov M., Ronneberger O., Tunyasuvunakool K., Bates R., Žídek A., Potapenko A. (2021). Highly accurate protein structure prediction with AlphaFold. Nature.

[31] Chen L., Fan Z., Chang J., Yang R., Hou H., Guo H., Zhang Y., Yang T., Zhou C., Sui Q. (2023). Sequence-based drug design as a concept in computational drug design. Nat. Commun..

[32] Kertesz M., Iovino N., Unnerstall U., Gaul U., Segal E. (2007). The role of site accessibility in microRNA target recognition. Nat. Genet..

[33] Manakov S.A., Shishkin A.A., Yee B.A., Shen K.A., Cox D.C., Park S.S., Foster H.M., Chapman K.B., Yeo G.W., Van Nostrand E.L. (2022). Scalable and deep profiling of mRNA targets for individual microRNAs with chimeric eCLIP. bioRxiv.

[34] Gu A., Dao T. (2024). Mamba: Linear-Time Sequence Modeling with Selective State Spaces. arXiv.

[35] Shazeer N. (2020). GLU variants improve Transformer. arXiv.

[36] Huang L., Zhang H., Deng D., Zhao K., Liu K., Hendrix D.A., Mathews D.H. (2019). LinearFold: Linear-time approximate RNA folding by 5’-to-3’ dynamic programming and beam search. Bioinformatics.

[37] Garcia D.M., Baek D., Shin C., Bell G.W., Grimson A., Bartel D.P. (2011). Weak seed-pairing stability and high target-site abundance decrease the proficiency of lsy-6 and other microRNAs. Nat. Struct. Mol. Biol..

[38] Fedus W., Zoph B., Shazeer N. (2022). Switch Transformers: Scaling to Trillion Parameter Models with Simple and Efficient Sparsity. J. Mach. Learn. Res..

[39] Wieder O., Kohlbacher S., Kuenemann M., Garon A., Ducrot P., Seidel T., Langer T. (2020). A compact review of molecular property prediction with graph neural networks. Drug Discov. Today Technol..

[40] Kipf T.N., Welling M. (2017). Semi-Supervised Classification with Graph Convolutional Networks. arXiv.

[41] Hamilton W.L., Ying R., Leskovec J. (2017). Inductive Representation Learning on Large Graphs. arXiv.

[42] Brody S., Alon U., Yahav E. (2022). How attentive are graph attention networks?. arXiv.

