# Supplementary material for: DeepExoMir: A Reproducible RNA Language Model Framework for CLIP-Seq-Supported MicroRNA Target-Site Prioritization
Source: Int J Mol Sci. 2026 Jul 10;27(14):6184. doi: 10.3390/ijms27146184 (PMC13410109; doi:10.3390/ijms27146184)
Supplement: Supplementary file 1 [file ijms-27-06184-s001.zip › ijms-4350516-supplementary.pdf]

Supplementary Information

DeepExoMir: A Reproducible RNA Language Model Framework for CLIP-seq-Supported MicroRNA Target-Site Prioritization

Wen-Hsien Lin[1,*], Chia-Ni Hsiung[1], Wen-Yu Lien[2], Martin Sieber[3]

[1]AI and Data Applications Division, GGA Corp., Taipei 114065, Taiwan

[2]BIONET Therapeutics Corp., Taipei 114065, Taiwan

[3]BIONET Corp., Taipei 114065, Taiwan

[*]Corresponding author: BryceLin@bionetTX.com

Contents

S1 Per-feature permutation importance (v14alt2L diagnostic) 3

S2 Retrospective-validation control experiments (full detail) 4
- S2.1 (i) miRNA-permutation control . . . 4
- S2.2 (ii) Random-score negative control . . . 4
- S2.3 (iii) Random gene-set control . . . 4

S3 DeepExoMir model architecture (full layer-by-layer specification) 5
- S3.1 Glossary of custom module names . . . 5
- S3.2 Input projection . . . 5
- S3.3 Hybrid encoder (8 layers) . . . 5
- S3.4 Interaction pooling . . . 5
- S3.5 Biological feature encoders (parallel) . . . 6
- S3.6 DuplexGAT module (v22 variant only) . . . 6
- S3.7 MoE classifier and multi-task heads . . . 6

S4 Retrospective validation methodology (full procedural detail) 7
- S4.1 Test miRNA panel . . . 7
- S4.2 Pigmentation gene-set . . . 7
- S4.3 Training-data leakage analysis . . . 7
- S4.4 Transcriptome-scale target scoring . . . 8
- S4.5 Scoring: v19 main (primary) and v19_no15b (miR-15b-5p sensitivity only) . . . 8
- S4.6 Pipeline correctness verification . . . 8
- S4.7 Per-gene aggregation . . . 9
- S4.8 Comparison baseline (TargetScan 8.0) . . . 9
- S4.9 KEGG pathway enrichment . . . 9

S5 v19_no15b retraining sensitivity analysis 10
- S5.1 Motivation . . . 10
- S5.2 Procedure . . . 10
- S5.3 Results on Hejret/Klimentova/Manakov benchmark . . . 10
- S5.4 Results on *hsa-miR-15b-5p* retrospective validation . . . 10

S6 Multi-seed stability — full table 11

S7 Retrain-from-scratch ablations (full detail) **12**
S7.1 Ablation implementation 12
S7.2 Training configurations 12
S7.3 Training outcomes 12
S7.4 Per-test-set evaluation (best-val checkpoints) 12
S7.5 Paired sample-level bootstrap 12
S7.6 Cross-probe consistency 13
S7.7 Val/test discrepancy note for v19_noStructure 13
S7.8 Reproducibility 14

S8 Implementation and availability summary **15**

S9 Companion paper: scope and content delineation **16**

This Supplementary Information accompanies the main manuscript and provides extended methodological detail and control experiments that were summarized in the main text for length reasons. All data, code, and full experimental outputs are deposited at:

- Source code: `https://github.com/linwenh09/DeepExoMir`
- Pre-trained checkpoints + diagnostics: Zenodo DOI `10.5281/zenodo.19216306`

S1. Per-feature permutation importance (v14alt2L diagnostic)

To complement the inference-time feature-masking ablation reported in Figure 3A of the main text, we performed permutation importance analysis on an earlier 31-feature variant (v14alt2L) on a held-out validation subset (50,000 samples, 3 repeats per feature). Each biological feature was independently shuffled and the resulting AU-PRC drop measured.

Why AU-PRC here, and not ROC-AUC? Permutation importance is an inference-time-style diagnostic on a specific trained checkpoint, so we use the inference-time metric (AU-PRC) used by miRBench convention and by Figure 3A of the main text. This differs from the ROC-AUC convention used for development-time progression in Table 2 and Figure 3B.

Aggregate findings (summed single-feature permutation importance, normalized over features with positive importance only — legacy pairing-statistics features were excluded from the normalization because they contributed negligible or slightly negative importance; the remaining three categories sum to ≈100%):

- Five conservation/site-location features (PhyloP mean, max, seed-mean, site_in_3′UTR, site_in_cds): ∼52%
- Twelve thermodynamic features (including ensemble MFE and duplex MFE): ∼42%
- Six ViennaRNA accessibility features: ∼6%
- Eight legacy pairing-statistics features: negligible or slightly negative (excluded from the normalization) — directly motivated their pruning in v16a/v18/v19

Conceptual note: permutation injects out-of-distribution noise into the input, whereas the inference-time zeroing in main-text Figure 3A sets features to values consistent with missing-data training examples. The two probes are therefore complementary, and we report both.

Per-feature numerical values are deposited in the Zenodo archive (`supplementary/v14alt2L_permutation_`

S2. Retrospective-validation control experiments (full detail)

The main text reports a compact summary of three control analyses ruling out gene-universe confounds. Full per-control details follow. Each control asks whether the observed rank-recovery signal could arise from the gene universe alone rather than from genuine miRNA-target specificity: the miRNA-permutation control breaks the miRNA-to-prediction mapping, the random-score control replaces the model scores with random values, and the random gene-set control replaces the curated pigmentation set with size-matched random genes. A genuine signal should survive the first two, and may legitimately fail the third when the reference set is coarse.

S2.1 (i) miRNA-permutation control

We shuffled the miRNA-to-predictions mapping and re-tested literature-target rank recovery across 100 random permutations.

Observed median literature-target percentiles (14.4–35.2%) were significantly better than the permuted distributions:

- *hsa-miR-203a-3p*: empirical $p < 0.001$
- *hsa-miR-139-5p*: empirical $p < 0.001$
- *hsa-miR-15b-5p*: empirical $p < 0.001$
- *hsa-miR-126-3p*: empirical $p = 0.057$

This demonstrates that target recovery is miRNA-specific rather than an artifact of the gene universe used for evaluation.

S2.2 (ii) Random-score negative control

For a given miRNA, KEGG pathway enrichment of its top-500 score-ranked predictions was compared to that of 500 randomly-selected predictions.

For *hsa-miR-6862-5p* (Tier-1 novel, effectively unseen during training), the Melanogenesis pathway:

- Top-500 score-ranked: rank 7 among KEGG pathways tested ($p = 0.013$)
- Random-500: rank 201 ($p = 0.72$)

This dramatic difference confirms that score-based ranking, rather than any gene-universe property, drives the pathway signal.

S2.3 (iii) Random gene-set control

Top-500 overlap with our 57-gene pigmentation list was compared to 100 random size-57 gene sets.

- *hsa-miR-203a-3p*: observed pigmentation fold-enrichment 1.01, exceeding 91% of random sets (empirical $p = 0.090$)
- Five Tier-1 novel miRNAs: pigmentation fold enrichment was *not* significantly above random (empirical $p \geq 0.32$ for all)

Interpretation of the negative result: a broad 57-gene pigmentation list is too coarse for fold-enrichment discrimination, since miRNAs regulate hundreds of genes broadly. This control's null result motivated our primary emphasis on (a) literature-validated targets (miRTarBase strong-evidence MTI), which pass the miRNA-permutation control at $p < 0.001$, and (b) KEGG pathway enrichment, where the Melanogenesis pathway hit for *hsa-miR-6862-5p* survives the random-score control.

S3. DeepExoMir model architecture (full layer-by-layer specification)

The main text provides a compact architecture summary. Full layer-by-layer specifications follow.

S3.1 Glossary of custom module names

The non-standard module and component names used in the main text and this Supplement are defined below.

RiNALMo-giga The 650M-parameter variant of the RiNALMo RNA language model, used as a frozen embedding backbone.

BiConvGate Bidirectional depthwise-separable convolution block with SwiGLU gating, for local sequence-pattern extraction.

Hybrid encoder Eight-layer stack alternating BiConvGate and cross-attention layers.

InteractionPool Multi-head attention pooling (4 heads) producing a 512-dimensional joint miRNA–target representation.

6-channel BP CNN Convolution over the 30×50 miRNA–target base-pairing matrix encoded in six channels.

DuplexGAT Duplex graph attention network (v22 variant only) over an 80-node nucleotide graph using GATv2Conv layers.

Multi-task heads Auxiliary regression heads (seed binding strength, duplex MFE, binding-site position) used to regularise training.

S3.2 Input projection

PCA-reduced RiNALMo embeddings (1280 → 256 dimensions, 88.7% variance retained) are projected through separate linear layers with layer normalization for miRNA (30 positions) and target (50 positions) sequences, followed by learnable positional embeddings.

S3.3 Hybrid encoder (8 layers)

The core encoder comprises 8 layers alternating between two layer types:

- **BiConvGate layers** (4 total): bidirectional depthwise separable convolution with SwiGLU gating. Hidden dimension 256, kernel size 5.
- **Cross-attention layers** (4 total, every 2 layers): inter-sequence information exchange through 8-head attention.

All layers incorporate pre-normalization, residual connections, and stochastic depth (drop path rate 0.1).

S3.4 Interaction pooling

A multi-head attention mechanism (4 heads) computes self-attention within each sequence and cross-attention between sequences, producing a 512-dimensional interaction vector that replaces standard mean pooling.

S3.5 Biological feature encoders (parallel)

Three parallel encoders process complementary biological inputs:

1. **6-channel base-pairing CNN**: encodes the 30×50 alignment matrix (channels: Watson-Crick, wobble, self-pair, mismatch, gap, position-aware). Output: 128-dimensional vector.
2. **2-layer structural MLP**: processes 33 pre-computed features spanning thermodynamic stability, ViennaRNA energetics, PhyloP conservation (5 features), and miRNA secondary structure. Output: 64-dimensional vector.
3. **Contact map CNN**: computes soft interaction maps from encoder outputs. Output: 128-dimensional vector.

S3.6 DuplexGAT module (v22 variant only)

The extended exploratory variant (v22) replaces the base-pairing CNN with a duplex graph attention network. The miRNA-target duplex is represented as a graph with 80 nucleotide nodes (30 miRNA + 50 target) connected by:

- Backbone edges (sequential connectivity)
- Watson-Crick / wobble base-pairing edges
- Proximity edges (within 2 nt)

Two GATv2Conv layers with 4 attention heads and learned edge-type embeddings produce a 128-dimensional graph-level representation.

Note: The main reported model v19 does *not* include the DuplexGAT module; duplex geometry is encoded via the 6-channel base-pairing CNN. The v19_no15b checkpoint is used only for the *hsa-miR-15b-5p* leakage-sensitivity analysis and shares the same architecture as v19 main. DuplexGAT (v22 exploratory variant) adds 1.5M parameters and increases per-epoch training time by approximately 65% due to graph construction overhead. For applications where inference speed is critical, the v19 architecture provides comparable validation performance (val ROC-AUC 0.852 vs 0.851 for v22) at substantially higher throughput.

S3.7 MoE classifier and multi-task heads

All features are concatenated (1216-dimensional total) and processed by a Mixture-of-Experts classifier with 4 expert networks, top-2 gating, and Platt scaling.

Auxiliary multi-task heads provide additional training signal:

- Seed binding strength regression
- Duplex MFE regression
- Binding site position prediction

S4. Retrospective validation methodology (full procedural detail)

The main text provides a compact summary of the retrospective validation pipeline. Full procedural details follow.

S4.1 Test miRNA panel

Nine experimentally characterized exosomal miRNAs from our companion study (Hsiung et al., 2026):

- Five novel "reduce melanin" miRNAs: *hsa-miR-6862-5p*, *-3622b-5p*, *-7847-3p*, *-6774-5p*, *-4685-5p*
- Four literature-known "enhance melanin" miRNAs: *hsa-miR-203a-3p*, *-126-3p*, *-139-5p*, *-15b-5p*

Mature miRNA sequences obtained from miRBase v22.1.

S4.2 Pigmentation gene-set

A list of 57 pigmentation-related genes was compiled from:

- KEGG hsa04916 (Melanogenesis)
- OMIM disease-gene associations for pigmentation disorders
- GO:0048770 (pigment granule organization)

This was supplemented with literature-curated master regulators (KITLG, MITF, TYRP1, SLC24A5, LEF1, CREB1, RAB27A, etc.).

S4.3 Training-data leakage analysis

To enable transparent retrospective validation, we performed direct string matching of the 9 miRNA identifiers against all rows of the miRBench training TSV files (AGO2 eCLIP Manakov2022 and AGO2 CLASH Hejret2023 training sets, totaling 1.9M pairs).

Per-miRNA training-row counts were used to classify miRNAs into four leakage tiers:

- **Tier 1 (clean)**: $\leq$6 training rows ($\leq$0.0003%)
- **Tier 2 (near-clean)**: 7–100 training rows
- **Tier 3 (moderate)**: 101–10,000 training rows
- **Tier 4 (contaminated)**: >10,000 training rows

Tier-4 miRNAs were excluded from primary generalization claims. Specifically, *hsa-miR-15b-5p* (51,586 training rows, 2.7% of training set) was reclassified as Tier-4 and addressed via a dedicated retraining sensitivity analysis (see §S5). The tier boundaries follow an order-of-magnitude scale, with the effectively-unseen Tier 1 set conservatively at the maximum exposure observed among the de-novo miRNAs in this panel (six rows); miRNAs appearing more often, even at low single-digit counts such as *hsa-miR-203a-3p* (seven rows), are placed conservatively in the next tier. The protocol generalises to other benchmarks: compute per-entity exact-match exposure counts against the training partition, bin them by order of magnitude, and run a retrain-without sensitivity check for any tier whose exposure could plausibly drive memorisation.

S4.4 Transcriptome-scale target scoring

We extracted 19,366 human (species_id=9606) 3′UTR sequences from the TargetScan 8.0 multi-species alignment file, stripping gaps and retaining the longest UTR per gene symbol.

For each miRNA, we identified canonical seed-matched sites by:

1. Computing the reverse complement of miRNA positions 2–7 (6mer core)
2. Searching for occurrences in each 3′UTR
3. Classifying sites as 8mer, 7mer-m8, 7mer-A1, or 6mer following TargetScan convention
4. Extracting a 50-nt target window centered on each seed match

This yielded 117,875 candidate (miRNA, site) pairs across all 9 miRNAs.

S4.5 Scoring: v19 main (primary) and v19_no15b (miR-15b-5p sensitivity only)

Each candidate pair was scored using the **v19 main checkpoint** (the same model benchmarked against the eight miRBench baselines in main-text Table 1). For *hsa-miR-15b-5p* only (the single Tier-4 contaminated miRNA), we additionally repeated the scoring with the **v19_no15b sensitivity checkpoint**, which was retrained from scratch after removing all 56,963 *hsa-miR-15b-5p*-containing rows from the full dataset (51,586 of which fall in the training split, the 2.7% exposure used for tier classification in §S4.3); see §S5.

Embedding computation: RiNALMo-giga backbone computed per-token embeddings on-the-fly. PCA reduction (1280→256) was applied using the parameters fit to the original training set.

Structural feature computation: 28 of 33 features were computed live from sequence at scoring time:

- All v7–v13 ViennaRNA-derived thermodynamic features
- v18 miRNA-asymmetry features
- v16c genomic-context features

Five PhyloP conservation features were set to zero because per-site BigWig lookups were not performed for de-novo-identified target windows. site_in_3′UTR was set to 1 by construction (all scored sites are 3′UTR windows).

Base-pairing matrix: A 6-channel base-pairing matrix was computed by nucleotide pair matching for each candidate window.

S4.6 Pipeline correctness verification

To verify that the retrospective scoring pipeline yields rankings consistent with the full-feature training pipeline, we re-scored the Hejret CLIP-seq test set using identical retrospective code:

- Retrospective pipeline: ROC-AUC = 0.823 / AU-PRC = 0.846
- Full-feature pipeline (Table 1 in main text): ROC-AUC = 0.830 / AU-PRC = 0.851
- Gap: <1% ROC-AUC, <0.5% AU-PRC

This small gap is consistent with the inference-time feature-masking ablation result (Figure 3A in main text), confirming that PhyloP conservation features contribute negligibly at the trained-model operating point. The retrospective predictions therefore closely reflect the model's full benchmark behavior.

Total scoring time: 485 seconds on a single RTX 5090 GPU at batch size 64.

S4.7 Per-gene aggregation

Predictions were aggregated per gene by taking the maximum score across all candidate sites in the 3′UTR. This conservative aggregation strategy emphasizes the strongest predicted binding site per gene.

S4.8 Comparison baseline (TargetScan 8.0)

TargetScan 8.0 default predictions were obtained from the file `Predicted_Targets_Context_Scores.default_`

Per-(miRNA, gene) scores were aggregated by taking the minimum (most negative) weighted context++ score across all sites for that gene. Predictions were filtered to the 9 miRNAs in our panel, including seed-variant suffixes (`.1`, `.2`).

S4.9 KEGG pathway enrichment

Enrichment for top-500 DeepExoMir predictions per miRNA was performed using Enrichr (KEGG_2021_Human library) via the gseapy Python package. Benjamini-Hochberg FDR correction was applied across all 90 hypothesis tests (9 miRNAs $\times$ 10 melanogenesis-relevant pathways).

S5. v19_no15b retraining sensitivity analysis

S5.1 Motivation

Direct string-matching analysis revealed that *hsa-miR-15b-5p* appears in 51,586 rows of the miRBench training set (2.7% of all training pairs). This placed it in our Tier-4 (contaminated) category. To verify that observed retrospective performance is not driven by training memorization, we performed a dedicated retraining sensitivity analysis.

S5.2 Procedure

A new checkpoint, designated v19_no15b, was trained from scratch using:

- Identical hyperparameters to the v19 main checkpoint
- Identical model architecture
- Modified training set: all 56,963 rows containing *hsa-miR-15b-5p* (or its variant suffixes) removed prior to training
- Same train/val/test split otherwise

S5.3 Results on Hejret/Klimentova/Manakov benchmark

Performance of v19_no15b on the held-out miRBench test sets:

- Hejret AU-PRC: 0.847 (vs 0.848 for v19 main)
- Klimentova AU-PRC: 0.866 (vs 0.868 for v19 main)
- Manakov AU-PRC: 0.846 (vs 0.848 for v19 main)

The minor performance drop ($\sim$0.002 AU-PRC) is within the multi-seed stability range, indicating the contaminating rows are not the source of overall benchmark performance.

S5.4 Results on *hsa-miR-15b-5p* retrospective validation

Using v19_no15b for retrospective scoring on *hsa-miR-15b-5p*:

- 20/22 literature-validated targets recovered (90.9%)
- Median percentile rank of literature targets: top-14.4%
- Permutation FDR-$q < 10^{-4}$

This confirms that the strong literature-target enrichment for this miRNA reflects genuine out-of-distribution generalization rather than training memorization.

S6. Multi-seed stability — full table

The main text reports the multi-seed mean and standard deviation. Full per-seed values follow:

Table S1: Multi-seed evaluation of v19 architecture (4 independent training runs).

**Seed**	**Val ROC-AUC**	**Test ROC-AUC**	**Test AU-PRC**
Original (default)	0.852	0.832	0.852
42	0.850	0.830	0.850
123	0.852	0.830	0.850
456	0.851	0.830	0.849
Mean $\pm$ SD	0.851 $\pm$ 0.001	0.831 $\pm$ 0.001	0.850 $\pm$ 0.001

The standard deviation across the four training runs is approximately 0.001 for all three metrics. This is roughly 40$\times$ smaller than the 0.04 AU-PRC gap over the best retrained CNN baseline, supporting the interpretation that the benchmark improvement is reproducible across training initializations.

S7. Retrain-from-scratch ablations (full detail)

This section provides complete procedural and numerical details for the three retrain-from-scratch ablations summarized in main-text Section 2.3.

S7.1 Ablation implementation

A `signal_ablation` keyword argument was added to `deepexomir.data.dataset.MiRNATargetDataset.__get` [illegible] When one of the three flags is set, the corresponding tensors are multiplied by zero at each data-loading call (both training and validation):

- **rnalm**: pooled and per-token RiNALMo embeddings for miRNA and target.
- **conservation**: five PhyloP-related positions in the 33-d structural-feature vector (phylop_mean, phylop_max, phylop_seed_mean, site_in_3′UTR, site_in_cds).
- **structure**: the 6-channel base-pairing matrix and the 28 non-PhyloP structural-feature positions.

S7.2 Training configurations

All three ablations used identical hyperparameters (`train_config_v19_noRNALM.yaml`, `_noConservation.yaml` [illegible] `_noStructure.yaml`), identical model architecture (`model_config_v19.yaml`), and the project's standard deterministic seed. Each variant trained for up to 60 epochs on the full 1.9M-pair miRBench training set, with early-stopping patience 20 (min_delta 0.0005 on val AUC) on an NVIDIA RTX 5090 GPU.

S7.3 Training outcomes

Table S2: Retrain-from-scratch ablation training outcomes.

Variant	Epochs run	Best val AUC	Wall time
v19_noRNALM	51 (early-stopped)	0.8410 @ epoch 42	13 h 50 min
v19_noConservation	48 (early-stopped)	0.8535 @ epoch 34	13 h 10 min
v19_noStructure	31 (early-stopped)	0.8238 @ epoch 10	10 h 54 min
v19 main (reference)	34 (reported)	0.8521	∼14 h

S7.4 Per-test-set evaluation (best-val checkpoints)

Each ablated checkpoint was then evaluated on the three miRBench test sets using the same `scripts/evaluate_mirbench.py` pipeline used for v19 main, with RiNALMo embeddings computed on-the-fly (the ablation flag continues to zero them for v19_noRNALM). Per-sample probability scores were saved for paired bootstrap.

S7.5 Paired sample-level bootstrap

Deltas versus v19 main were computed by paired sample-level bootstrap (10,000 resamples on Hejret/Klimentova, 2,000 on Manakov, seed 42). For each resample, AU-PRC was re-computed from the same bootstrapped indices for both the main-model scores and the ablated-model scores; two-sided p-values are reported from the bootstrap distribution of Δ.

Table S3: Retrain-from-scratch test-set performance. Mean AU-PRC is the arithmetic mean across the three datasets.

**Variant**	**Hejret**	**Klimentova**	**Manakov**	**Mean**
v19 main	0.848	0.868	0.848	0.855
v19_noRNALM	0.814	0.843	0.822	0.826
v19_noConservation	0.842	0.864	0.845	0.850
v19_noStructure	0.866	0.871	0.853	0.863

Table S4: Retrain-from-scratch ablation vs v19 main: paired bootstrap. Positive Δ means v19 main outperforms the ablation; negative Δ means the ablation outperforms v19 main. Significance: *** $p < 0.001$, ** $p < 0.01$, * $p < 0.05$, n.s. otherwise.

**Ablation**	**Test set**	**Main**	**Abl.**	Δ	**95% CI**	p
v19_noRNALM	Hejret	0.848	0.814	+0.035 **	[+0.014, +0.056]	0.0014
v19_noRNALM	Klimentova	0.868	0.843	+0.025 ***	[+0.011, +0.039]	0.0006
v19_noRNALM	Manakov	0.848	0.822	+0.026 ***	[+0.025, +0.027]	<0.001
v19_noConservation	Hejret	0.848	0.842	+0.007 n.s.	[−0.007, +0.021]	0.352
v19_noConservation	Klimentova	0.868	0.864	+0.004 n.s.	[−0.003, +0.011]	0.316
v19_noConservation	Manakov	0.848	0.845	+0.003 ***	[+0.003, +0.004]	<0.001
v19_noStructure	Hejret	0.848	0.866	−0.017 n.s.	[−0.037, +0.002]	0.075
v19_noStructure	Klimentova	0.868	0.871	−0.003 n.s.	[−0.014, +0.009]	0.640
v19_noStructure	Manakov	0.848	0.853	−0.004 ***	[−0.005, −0.004]	<0.001

S7.6 Cross-probe consistency

Both inference-time feature-masking (main-text Figure 3A) and retrain-from-scratch ablations agree on the qualitative ranking: RiNALMo is dominant; PhyloP is redundant; structure is approximately redundant. They disagree only on magnitude for RiNALMo (retrain −0.029 mean vs. inference-time −0.055 to −0.161), reflecting a trained model's heavier reliance on its final operating point than a fresh model's retrain-from-scratch dependence during optimization. This cross-probe asymmetry is itself methodologically informative: inference-time probes report a trained checkpoint's operating-point dependence, whereas retrain-from-scratch probes report a signal's training-time necessity within this architecture.

S7.7 Val/test discrepancy note for v19_noStructure

v19_noStructure shows an unusual val/test pattern. Its best val AUC (0.8238 @ epoch 10) is the lowest among the three ablation variants, yet its mean test AU-PRC (0.863) is the highest among them and even slightly exceeds v19 main's 0.855. This discrepancy is $\sim 40\times$ the multi-seed stability SD (0.001) and therefore not attributable to run-to-run noise. We interpret it as evidence that val-set-driven architecture selection during our v1→v19 iteration may have favored features (the 33 hand-crafted structural descriptors) that correlate with val-set distributions but do not transfer to the independent test sets. This methodological caveat applies broadly to miRNA target prediction development and suggests future papers should perform retrain-from-scratch ablations (or equivalent training-time dependence probes) rather than relying exclusively on val-set-level ablation evidence.

S7.8 Reproducibility

Full paired bootstrap CSV (ablation_all_variants.csv), Markdown interpretation (ablation_all_variants[illegible] and per-sample prediction score files ({dataset}_test_v19_{noRNALM,noConservation,noStructure}.npy) are provided in the project Zenodo archive.

S8. Implementation and availability summary

Table S5: DeepExoMir implementation summary.

Field	Value
Source code	`https://github.com/linwenh09/DeepExoMir` (MIT license)
Pre-trained checkpoints	Zenodo DOI: `10.5281/zenodo.19216306`
License	MIT (code), CC-BY 4.0 (model weights)
Required dependencies	Python 3.11, PyTorch 2.1, PyTorch Geometric 2.4, RiNALMo (frozen), ViennaRNA 2.5, NumPy, scikit-learn, gseapy
Hardware requirements	NVIDIA GPU for embedding generation ($\geq$16 GB VRAM; RTX 4090 / A100 recommended for training). Scoring from cached embeddings runs on smaller GPUs and is expected to be CPU-tractable for moderate jobs, though CPU-only inference was not formally profiled
Training time	$\sim$14 hours on RTX 5090 (v19, 55 epochs)
Inference time per (miRNA, target) pair	$\sim$0.2 ms (amortized, batch size 64, RTX 5090)
Inference throughput	$\sim$4,600 samples/sec
Embedding cache size (PCA-256, 1.9M pairs)	31 GB
Checkpoint size	106 MB (v19) / 112 MB (v22)
Benchmark dataset	miRBench v3 (Sammut et al., 2025)
Reproducible commands	`python scripts/evaluate_mirbench.py -checkpoint checkpoints/v19/...`
Test data + expected output	`tests/expected_outputs/` in repository
Containerized environment	Dockerfile + Conda environment.yml provided

Quick-start example (predict targets for a custom miRNA):

```
python scripts/predict_targets.py \
    --mirna-seq UAUUGCACUUGUCCCGGCCUGUUU \
    --utr-fasta my_3utr_sequences.fa \
    --checkpoint checkpoints/v19/checkpoint_epoch034.pt \
    --output predictions.tsv
```

S9. Companion paper: scope and content delineation

This IJMS submission is the methodology counterpart to our companion biological paper:

> Hsiung, C.-N.; Lien, W.-Y.; Sieber, M.; Lin, W.-H. *Omic Profiling of Extracellular Vesicles from Two Cord-Related Sources Reveals Divergent Effects on Melanogenesis.* Curr. Issues Mol. Biol. 2026, 48, 391. doi:10.3390/cimb48040391

The two manuscripts have non-overlapping content:

- **Companion paper (Hsiung et al., 2026)**: reports the experimental phenotypic effects of nine exosomal miRNAs on melanin production, including melanin assays in melanocyte cell culture, exosomal miRNA characterization, and biological interpretation of the pigmentation phenotype. The companion paper does *not* report computational predictions, benchmarking, or methodology development.
- **This IJMS submission**: reports the development, benchmarking, and retrospective validation of the DeepExoMir computational framework, including the four-tier leakage classification protocol, inference-time and cumulative-addition ablations, and per-tier retrospective validation against literature targets and pathway enrichment. This submission does *not* report new wet-lab experiments or biological phenotypes.

The shared element between the two manuscripts is limited to the panel of 9 miRNAs used as the retrospective validation benchmark in this IJMS submission and the experimental subjects in the companion paper. All computational analyses, baseline comparisons, leakage-tier disclosure framework, and benchmark evaluations in this IJMS submission are unique to it.
